# Selective exposure shapes the Facebook news diet

**DOI:** 10.1371/journal.pone.0229129

**Published:** 2020-03-13

**Authors:** Matteo Cinelli, Emanuele Brugnoli, Ana Lucia Schmidt, Fabiana Zollo, Walter Quattrociocchi, Antonio Scala

**Affiliations:** 1 Applico Lab, CNR-ISC, Rome, Italy; 2 Università di Venezia “Ca’ Foscari”, Venezia, Italy; 3 LIMS, the London Institute for Mathematical Sciences, London, United Kingdom; Central European University, HUNGARY

## Abstract

The social brain hypothesis approximates the total number of social relationships we are able to maintain at 150. Similar cognitive constraints emerge in several aspects of our daily life, from our mobility to the way we communicate, and might even affect the way we consume information online. Indeed, despite the unprecedented amount of information we can access online, our attention span still remains limited. Furthermore, recent studies have shown that online users are more likely to ignore dissenting information, choosing instead to interact with information adhering to their own point of view. In this paper, we quantitatively analyse users’ attention economy in news consumption on social media by analysing 14 million users interacting with 583 news outlets (pages) on Facebook over a time span of six years. In particular, we explore how users distribute their activity across news pages and topics. On the one hand, we find that, independently of their activity, users show a tendency to follow a very limited number of pages. On the other hand, users tend to interact with almost all the topics presented by their favoured pages. Finally, we introduce a taxonomy accounting for users’ behaviour to distinguish between patterns of selective exposure and interest. Our findings suggest that segregation of users in echo chambers might be an emerging effect of users’ activity on social media and that selective exposure—i.e. the tendency of users to consume information adhering to their preferred narratives—could be a major driver in their consumption patterns.

## Introduction

The social brain hypothesis approximates the total number of social relationships we are able to maintain at 150 [1, 2]. Such a theoretical cognitive limitation emerges in several other contexts [3] from the patterns of human mobility [4] to the way we communicate [5–8]. Furthermore, the uptake of social media has radically changed the way we consume content online. Indeed, the way we consume information and the cognitive limits and algorithmic mechanisms underpinning them has a bearing on foundational issues concerning our news consumption patterns. As a consequence, in 2017 the World Economic Forum issued a warning on the potential of social media to distort the perception of reality [9]; possibly, such risk is related to the fact that social media has induced a paradigm shift in the way we consume information [10, 11]. In a similar vein, recent studies targeting Facebook [12–14] have shown that content consumption is dominated by selective exposure [15–17]—i.e. the tendency of users to ignore dissenting information and to interact with information adhering to their preferred narrative – and that individual choices more than algorithms [14] also characterise the consumption patterns of users and their friends [18]. Users who display selective exposure tend to focus their attention on the information provided by a limited number of sources (e.g. news outlets) despite being aware of the presence of a wide array of alternatives.

Selective exposure may lead to the emergence of echo chambers [19, 20]—i.e. groups of like-minded people cooperating to frame and reinforce a shared narrative—thus facilitating fake news and more generally misinformation cascades [21, 22]. This is especially valid when considering the way in which we have shifted from a paradigm where information was supplied by few official news sources mediated by experts and/or journalists, to the current disintermediated environment composed by a heterogeneous mass of information sources. Social media play a pivotal role not only in our social lives, but also in the political and civic world, developing to such an extent that they have rapidly become the main information source for many users [23]. Essentially, online confirmation bias seems to account for users’ decisions about consuming and spreading content; at the same time, the aggregation of favoured information within those communities reinforces selective exposure and group polarisation [24, 25].

Several works have addressed the dynamics of news consumption through social media [26–28] and have explored the interplay between selective exposure and political polarisation on the Internet [29, 30]. Focusing on news consumption on social media, in [31] the authors find that users’ consumption patterns seem to determine the emergence of a sharp community structure among news outlets. Nowadays, the understanding of the impact of social media on the news business model is one of the most pressing challenges for both science and society [32–34].

In this paper, we perform a thorough quantitative analysis to characterise users’ attention dynamics on news outlets on Facebook. In particular, we study how 14 million Facebook users distribute their activity among 50000 posts, clustered by topics, produced by 583 pages (news outlets) listed by the Europe Media Monitor over a six-year time span. The downloaded data from each page include all of the posts made from 1 January 2010 to 31 December 2015, as well as all of the likes and comments on those posts (for further details refer to Materials and Methods). We find that users, independently of their activity and of the time they spend online, show a tendency to interact with a very limited number of news outlets. To test the presence of selective exposure, for which evidence emerges from users focusing their attention on a set of preferred news sources, we analyse how homogeneously users distribute their activity across pages and topics. More precisely, the concentration of the distribution of likes towards a certain page or topic signals the presence of selective exposure, while the heterogeneity of such a distribution determines the strength of selective exposure. We find that highly engaged users tend to concentrate their activity on few pages while being less selective regarding the topics presented by the pages. In general, we observe that selective exposure increases in strength when the activity of users (i.e. the number of likes) grows but is not affected by users’ lifetime (i.e. the time span between the first and the last like). Finally, we provide a taxonomy to classify users by means of their consumption patterns. Our results suggest that the tendency of users to limit their attention to a smaller number of news sources might be one of the factors behind the emergence of echo chambers online. The emerging outcome still underlines the tendency of users towards segregation, partly because of their attitude and cognitive limits, and partly because of the features of the social media in which they operate.

The paper is structured as follows. First, we describe the way users interact with posts, pages and topics, characterising their news consumption habits. Then, we analyse users’ attention patterns on topics and pages and discuss the mechanism of selective exposure as a quantitative heterogeneity problem. Finally, we conclude the paper by outlining a taxonomy of the users based on the comparison between their attention patterns with respect to pages and topics.

## Results and discussion

### Users’ news consumption

News appears on Facebook as posts, and users can interact with such posts through different actions, namely likes, comments and shares. A like is usually a positive feedback on a news item. A share indicates a desire to spread a news item to friends. A comment can have multiple features and meanings and can generate collective debate. Since our aim is to investigate the mechanism of selective exposure, we focus our analysis on the likes of the users (likes reactions), i.e. on their positive feedback towards certain posts. As shown in previous works [31], likes are a good proxy of the users’ activity in terms of engagement and attention patterns.

The interaction between users and posts can be represented as a bipartite network *G*_*up*_, undirected and unweighted, in which the first partition has *n*_*u*_ elements (corresponding to the users) while the second partition has *n*_*p*_ elements (corresponding to the posts). The matrix *I*_*up*_ representing such bipartite network is binary since a user is allowed to put one like per post; thus, we have *I*_*up*_ = 1 if user *u* likes post *p*, 0 otherwise. Given *G*_*up*_, the activity—i.e. the number of likes—of the user *u* can be quantified by his/her degree $k_{u}=\sum_{p=1}^{n_{p}}I_{up}$.

In order to investigate the relationship between user and pages, from the bipartite network *G*_*up*_ we obtain a second bipartite network with *n*_*u*_ users and *n*_*P*_ pages called $G_{uP}^{*}$, in which posts are simply grouped by the page that generated them. On such a network, the activity of the user remains unchanged and the number of likes of user *u* to page *P* can be obtained as $I_{uP}^{*}=\sum_{p\in P}I_{up}$.

Additionally, the posts of the user-post network *G*_*up*_ can be also grouped by the topic they treat using a topic modeling algorithm [35] as described in Materials and Methods. Aggregating *G*_*up*_ by topic, we generate a third bipartite network called $G_{ut}^{†}$ with *n*_*u*_ users and *n*_*t*_ topics. A post can be considered a mixture of topics, all appearing in a certain proportion, and the weighted bipartite network $G_{ut}^{†}$ is represented by the matrix $I_{ut}^{†}$ in which the weight of each element is proportional to the overall presence of a certain topic in the posts liked by a certain user. Using $I_{ut}^{†}$ we can study the activity of users with respect to different topics.

**Fig 1:**
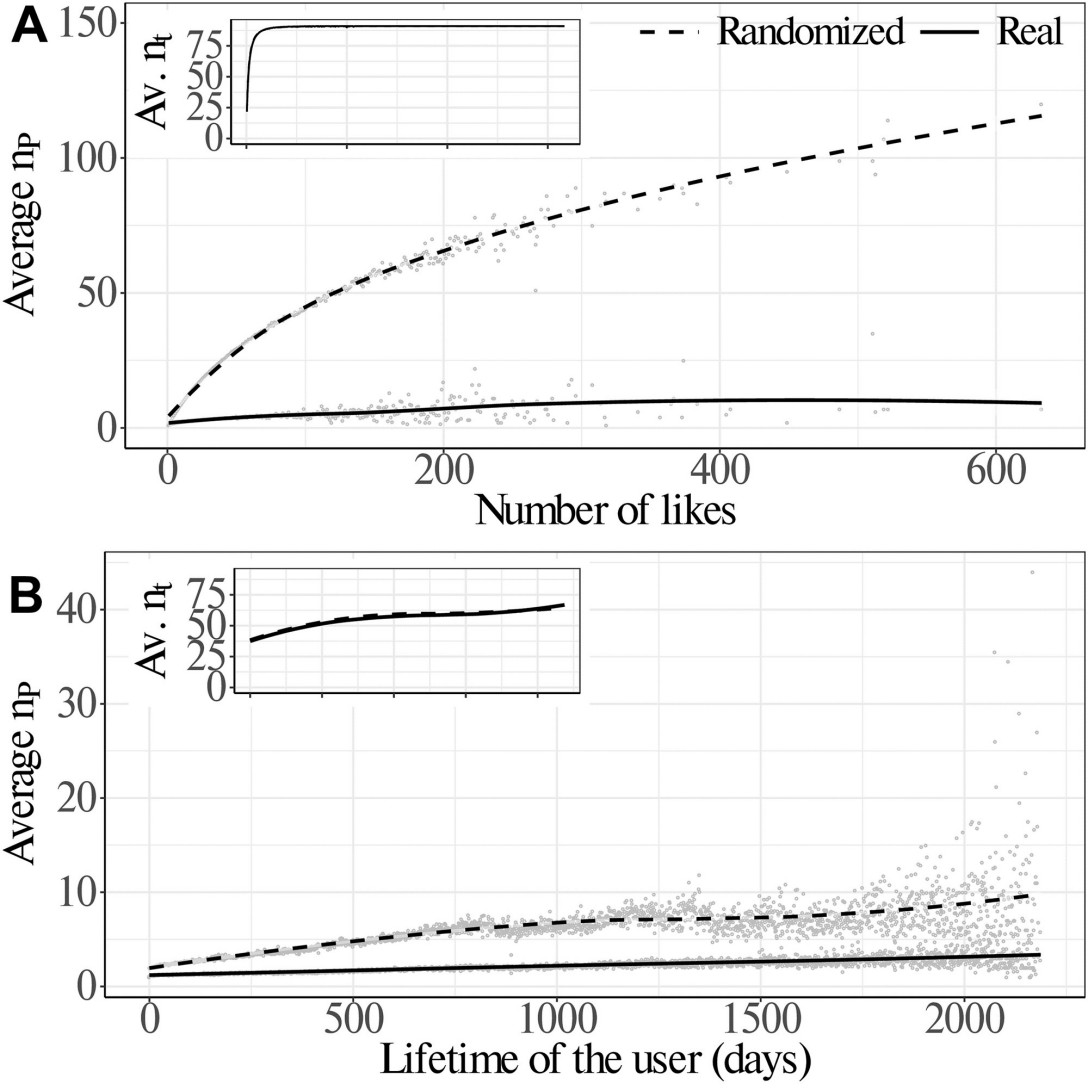
Correlations between users’ activity/lifetime and user engagement with pages and topics. Panel A: relationship between the average number of pages that received likes by users with respect to their activity (quantified by the number of likes). We observe that the average number of pages reaches a plateau of ∼10 pages for users with an activity of more than ∼300 likes. The dashed line represents the same analysis in the case of randomized data. In the inset of panel A, we show the relationship between the average number of topics covered by users with respect to their activity. We observe that users with an activity of more than ∼10 likes already reach a plateau corresponding to the overall number of topics that is 91 (as explained in Materials and Methods). Panel B: relationship between the average number of pages that received likes by users with respect to their lifetime (quantified by the time between the first and the last like). We observe that the average number of pages grows slowly and reaches a value of ∼3 pages for most lifelong users. The dashed line represents the same analysis in the case of randomized data. The inset of panel B, we show the relationship between the average number of topics and the users’ lifetime. We observe that for lifetime larger than ∼1000 days the number of topics reaches a value of ∼50, corresponding roughly to 50% of the overall topics. The curves are obtained by means of a loess regression.

Fig 1 shows the average number of pages liked by users with respect to their activity and lifetime; the former is defined as the number of likes of the user, whilst the latter is defined as the time span between the first and the last like a user put on two different posts. In Fig 1(A) we observe the relationship between the users’ activity and the number of pages they interact with. We notice that the average number of pages liked by a user reaches a plateau with increasing activity; in particular, users with more than ∼300 likes concentrate, on average, their activity on only ∼10 pages (for further details, see S1 File). This may be due to different—and possibly co-interacting—factors, such as the different narratives adopted by the pages in order to report information, the presence of natural limits to attention of the users, or even the filtering due to the ranking algorithms used in the information search.

To define the topics of the posts, we first pre-process the posts to extract the set of meaningful words *W* (see Material and methods) and then define the bipartite network $G_{pw}^{⋄}$ that links each post *p* to the words *w* used in the post. We then apply the hierarchical stochastic block-modeling algorithm of [35] (a well-assessed topic modeling algorithm that takes a bipartite network as input) on $G_{pw}^{⋄}$ to detect the topics and find 91 different topics. We observe that, since the analysed pages are news outlets, most pages tend to cover almost all the topics (see Fig 1 of S1 File).

The inset in Fig 1(A) shows the number of topics a user interacts given his/her activity. Different from what is observed in the interaction with pages, users tend to interact with many topics regardless of their activity. In particular, users with more than ∼10 likes already tend to interact with almost all the topics. Such interaction patterns could be explained by assuming that users tend to interact with all the topics presented by their preferred pages.

**Fig 2:**
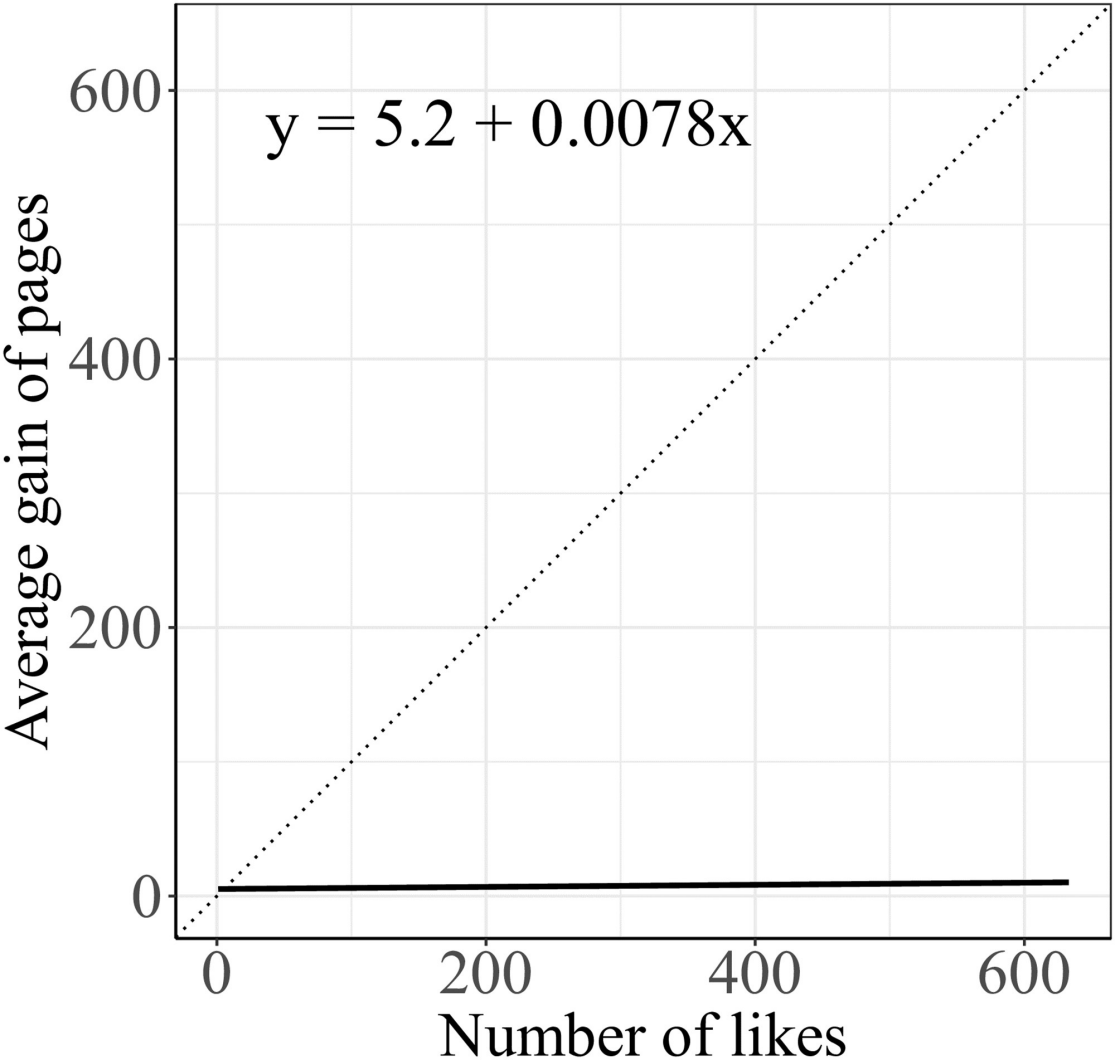
Average gain of pages for each new like of the user. The average gain of pages is displayed by means of a linear regression with equation *y* = 5.2 + 0.0078*x* and *r*^2^ = 0.348. The dotted line represents the case of linear growth.

In Fig 1(B) we notice that the average number of pages that users interact with grows slowly with the users’ lifetime. However, the average number of topics reaches a plateau corresponding to more than 50% of the overall topics for users with a lifetime larger than ∼1000 days. Additionally, we compared the average number of pages given the activity and the lifetime of the user with the same quantity after a randomization of the data. The randomization reshuffles the liking patterns of users while keeping their activity and lifetime. As a result of such a process the user is allowed to interact with a different set of topics and pages. The results are shown in Fig 1. We note that the average number of pages is always higher in the case of randomized data (dashed line) with respect to both activity and lifetime. Such aspects indicate that the average number of pages whose posts received likes by users is somewhat limited with respect to the value observed in the random case. In other words ‘unbiased’ users, i.e. those resulting from the randomization, tend to consume news from a wider amount of pages. In order to provide further empirical evidence for the selective choice of news outlets, we compute the average gain in terms of new pages per each new like of the users. By using a linear regression to interpret the data, we obtain the following equation: *y* = 5.2 + 0.0078*x* with an *r*^2^ = 0.348. The tiny value of the coefficient that is ∼0 supports the hypothesis of saturation in the number of pages on which the user is active. In Fig 2 the results of the obtained linear regression are compared with the case of linear growth with equation *y* = *x*. We note an almost constant trend of the growth in terms of gain of new pages.

### Attention patterns on topics

Selective exposure relates to the tendency of users to concentrate their activity on specific topics or pages while ignoring other ones. For instance, a user who focuses his/her activity on a single topic (or page) would display higher selective exposure than a user who interacts with multiple topics. Focusing on a single topic rather than on different ones entails a heterogeneity in the distribution of the user’s activity that can be directly associated with the mechanism of selective exposure.

Therefore, a good proxy for selective exposure is a measure that quantifies heterogeneity in the distribution of users’ activity across different elements; namely, topics or pages.

The Gini index [36] is a classic example of a synthetic indicator used for measuring inequality of social and economic conditions [37]; hence, to give a measure of selective exposure with respect to topics, we apply the Gini index (described in Material and methods) on the users’ activity on different topics as stored on the rows of the weighted incidence matrix $I_{ut}^{†}$. Notice that, consistently with the use of a state-of-the-art topic modeling algorithm [35], a post is considered a mixture of topics all appearing in different proportions. Consequently, the interaction of a user with multiple topics, which derives from liking one or more posts treating such topics, is still consistent with a mixed membership model [38].

We estimate the strength of selective exposure of user *u* to topics using the following expression of the Gini index:

$$\begin{array}{c} g^{†}=\frac{1}{2\;n_{t}}\frac{\sum_{t=1}^{n_{t}}\sum_{q=1}^{n_{t}}\left|I_{ut}^{†}-I_{uq}^{†}\right|}{\sum_{t=1}^{n_{t}}I_{ut}^{†}} \end{array}$$ (1)

**Fig 3:**
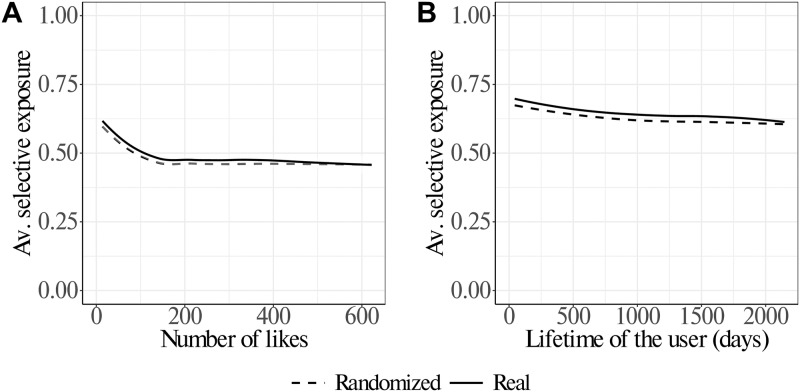
Average selective exposure of users (as measured by the Gini coefficient *g*^†^) with respect to their activity/lifetime. Results of the randomization of the actual data are reported as dashed lines. Panel A: average values of selective exposure to topics with respect to users’ activity show that increasing activity levels correspond to lower selective exposure, i.e. users concentrate on a higher number of topics. Panel B: average values of selective exposure to topics with respect to users’ lifetime (measured in days) show that the mechanism of choice of topics does not seem to be influenced by the time users have been present on the social medium. The average values of selective exposure are somewhat replicated after randomizing the liking patterns of users. The curves are obtained by means of a loess regression. Further details are reported in Fig 2 of S1 File.

Values of $g_{u}^{†}∼1$ signal that the user *u* concentrates his/her activity on few topics, while values of $g_{u}^{†}∼0$ signal the tendency to be active on different topics. The panels of Fig 3, show the strength of selective exposure (as measured by the Gini index $g_{u}^{†}$) with respect to the users’ activity and lifetime respectively. On the one hand, we observe that increasing values of activity correspond to a progressively weaker selective exposure; on the other hand, users’ lifetime does not show strong correlations with their focus on specific topics. This result is consistent with the fact that Facebook pages tend to span several topics (see Fig 1 of S1 File) and that highly active users are more likely to consume a wider range of topics, thus decreasing their selective exposure. In fact, even if users never get to a balanced “diet” of topics (corresponding to a Gini index ∼0), we note that users consume more topics with increasing activity, i.e. the most active users are those with the weaker selective exposure to topics. The dashed lines in the panels of Fig 3 display the average values of selective exposure after randomizing the liking patterns of users while keeping their activity and lifetime. As a result of such a randomization process the user is allowed to interact with a different set of topics and pages. We observe minor differences between the curves of Fig 3 concluding that the observed values of selective exposure to topics is reproducible by means of random consumption of posts. In order to compare the distribution of the Gini coefficient deriving from real data with the distribution deriving from randomized data we ran a Kolmogorov-Smirnov (KS) test. The test measures the similarity between two distributions in a non parametric way by comparing their cumulative distribution functions. It uses as test statistic the variable *D* ∈ [0, 1] that is the the maximum absolute difference between the cumulative distribution functions. A value of *D* = 0 means that the two distributions are the same. The KS test returns *D* = 0.1091 with *p* < 10^−5^ implying a small, yet significant, difference between the real and the randomized distributions of the Gini coefficient. In general, we note that users proportionally to their activity tend to span the topics covered by the pages (news outlets) they are active on, being far from strong topical selectivity (see also Fig 3 in S1 File).

### Attention patterns on pages

To understand whether the mechanism of selective exposure to pages—if present—could be different from that observed for topics, we replicate the analysis of the previous section by considering the matrix $I_{uP}^{*}$, i.e. considering the interaction of users with pages (news outlets).

In this case, the expression for the Gini index *g*_*u*_ of the user *u* with respect to pages he/she likes is:

$$\begin{array}{c} g^{*}=\frac{1}{2\;n_{P}}\frac{\sum_{P=1}^{n_{P}}\sum_{Q=1}^{n_{P}}\left|I_{uP}^{*}-I_{uQ}^{*}\right|}{\sum_{P=1}^{n_{P}}I_{uP}^{*}} \end{array}$$ (2)

**Fig 4:**
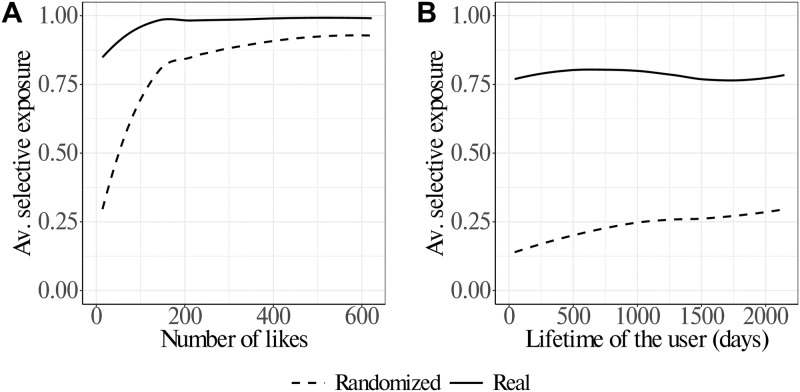
Average selective exposure of users (as measured by the Gini coefficient *g*^†^) with respect to their activity/lifetime. Results of the randomization of the actual data are reported as dashed lines. Panel A: average values of selective exposure to pages with respect to users’ activity show that increasing activity levels correspond to higher selective exposure, i.e. users concentrate on fewer pages. Panel B: average values of selective exposure to pages with respect to users’ lifetime display an oscillatory trend. The average values of selective exposure cannot be replicated after randomizing the liking patterns of users. The curves are obtained by means of a loess regression. Further details are reported in Fig 5 of S1 File.

However, applying the Gini index to our dataset would introduce a bias due to the sparsity of the matrix $I_{uP}^{*}$. In fact, we have many users whose activity is smaller than the number of pages (i.e. the sum of the entries of a row *u* of I* is often much smaller than the number of columns *n*_*P*_). In such cases, the Gini index displays a bias towards high values [39] of g* (see Fig 4 of S1 File) since the denominator of Eq 2 is small and the possibility of perfect equidistribution—i.e. the same number of likes on each page—cannot be achieved. Therefore, to avoid such a flaw of the Gini index in the case of sparse data, we renormalise the Gini index according to the minimum and maximum values it can assume:

$$\begin{array}{c} g^{⊳}=\frac{g^{*}-g_{min}^{*}}{g_{max}^{*}-g_{min}^{*}} \end{array}$$ (3)

where $g_{max}^{*}=1$ is the maximum value of the Gini coefficient, while $g_{min}^{*}$ is the minimum value of the Gini coefficient. As shown in Materials and Methods, $g_{min}^{*}$ depends on the number of likes *n*_*l*_ and on the number of pages *n*_*P*_; when *n*_*l*_ < *n*_*P*_, due to the “not enough data bias” we have that $g_{min}^{*}>0$. Thus, the quantification of selective exposure can be assessed using the normalised Gini index *g*^⊳^ as in Eq 3.

In the top panels of Fig 4 we observe that the mechanism of selective exposure is present also in the case of pages, but with a completely different trend than what is observed in the case of topics. On the one hand, we observe that increasing values of activity correspond to a concentration of users toward high values of $g_{u}^{⊳}$, i.e. users’ selective exposure to pages increases. On the other hand, users’ lifetimes do not show strong correlations with $g_{u}^{⊳}$; hence, the mechanism of choice of pages does not seem to be influenced by the time users has been present on the medium. Such results are consistent with a way of choosing pages (news outlets) based on selective exposure rather than on a comparison among several sources; it is also consistent with a reinforcement mechanism for which the higher the activity, the stronger the concentration on fewer pages. In other words, we observe that users, especially the most active, tend to affiliate with pages and narratives regardless of the topics they treat. What appears is that the consumption of news depends on very few sources of information and could be almost independent of the subjects treated.

The dashed lines in the panels of Fig 4 display the average values of selective exposure after randomizing the liking patterns of users while keeping their activity and lifetime. We observe increasing selective exposure when we take into account the activity of the user while we observe a more oscillatory trend in the case of lifetime. Therefore, the lifetime is likely to play a different, and perhaps negligible, role for the trend of selective exposure when compared to activity. Indeed, having a long lifetime doesn’t necessarily imply having a high activity since the two quantities are positively but not perfectly correlated. The observation of growing selective exposure in the random case may be due to the fact that the activity of pages is heterogeneous [40], some pages produce many more posts than others, and therefore more active users are likely to end up consuming posts from such active pages even in the random case. In general, selective exposure in randomized data is weaker than in real data where the consumption of contents from few news outlets (i.e. selective exposure) seems to derive from deliberate actions of the users, as observed in [14], rather than from random choices. In order to compare the distribution of the Gini coefficient deriving from real data with the distribution deriving from randomized data we ran a Kolmogorov-Smirnov (KS) test. The KS test returns *D* = 0.9125 with *p* < 10^−5^ implying a significant difference between the real and the randomized distributions of the Gini coefficient.

### Comparing activity on pages and topics

In this section we compare the two mechanisms of selective exposure. Indeed, users can display different profiles of selective exposure with respect to pages and topics, and the knowledge of both dimensions can be helpful in order to characterise their attention patterns on social media.

**Fig 5:**
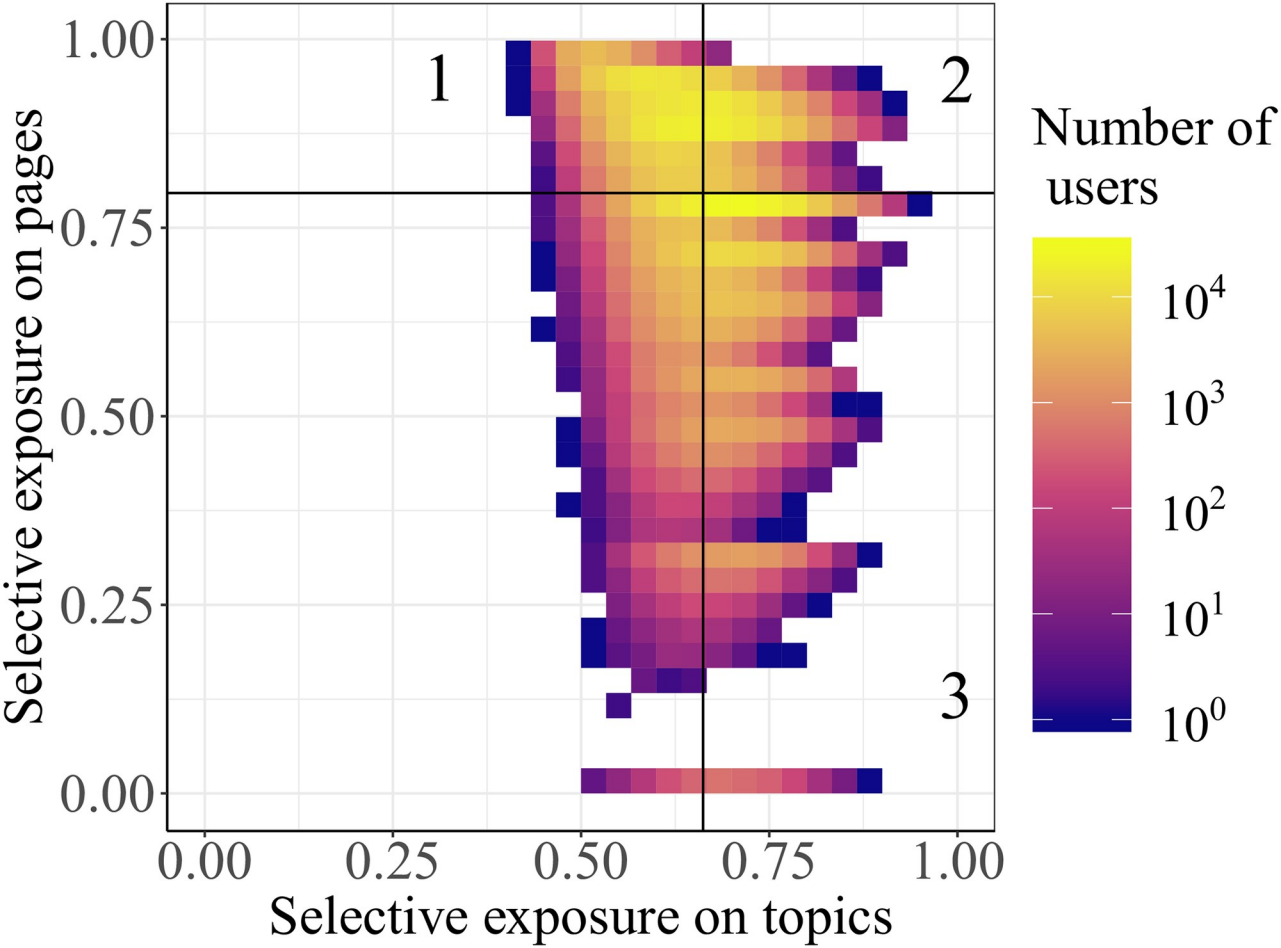
Interrelation between the mechanism of selective exposure to pages and topics. The area is divided in four regions determined by the average values of selective exposure to topics *g*^†^ = 0.662 and topics *g*^⊳^ = 0.796. The distributions of *g*^†^ and *g*^⊳^ are reported in Fig 6 of S1 File. Three out of four regions are labelled since they can be associated with different kind of selective exposure displayed by users. The region of multi-topic selective exposure is located in the top-left, the region of single-topic selective exposure is located in the top-right while the region of exposure by interest is located in the bottom left. The colour scale of the distribution represents the number of users related to a certain (*x*, *y*) couple.

In Fig 5, by combining the results related to users’ selective exposure to both pages and topics, we report different classes of users based on their statistical signatures. Users can be classified in three classes that are related to a specific type of selective exposure:

1. *Multi-topic selective exposure*: high selective exposure to pages and low selective exposure to topics. Users in the region of multi-topic selective exposure are affiliated with one or few pages while spanning many topics.
2. *Single-topic selective exposure*: high selective exposure to pages and high selective exposure to topics. Users in the region of single-topic selective exposure are affiliated with one or few pages but they tend to focus their attention on specific content.
3. *Exposure by interest*: low selective exposure to pages and high selective exposure to topics. Users in the region of interest are not affiliated with pages but browse different sources while consuming the content they are interested in.

Fig 5 is divided in four regions determined by the average values of selective exposure to pages and topics.

The region of multi-topic selective exposure is located in the top-left, the region of single-topic selective exposure is located in the top-right while the region of exposure by interest is located in the bottom right. In Fig 5 we observe that a large fraction of users are located in the region of multi-topic selective exposure, accordingly with the fact that users tend to display a high selective exposure to pages and a low selective exposure to topics. The users with highest selective exposure to pages are also those with the highest activity. Other users are located in the region of single-topic selective exposure meaning that they tend to focus on few pages and topics. We note that such users, having a high selective exposure to topics, also display an average activity that is lower than that of users in the region of multi-topic selective exposure (see Fig 3).

The region of exposure by interest is well populated; however, in such a region (as well as the fourth region located in the bottom left) the characterisation of the behaviour has to be carefully considered, since users users with low selective exposure to pages are also those with the lowest activity (see Figs 3 and 4).

## Conclusions

In this paper we explored the users’ news diet on social media. The economy of attention on social media is characterised by different features, one of which is selective exposure. Analysing the interaction between 14 millions users and 583 news outlets, we find that on average users tend to interact with a very limited number of pages and that, similarly to a Dunbar number, this aspect weakly depends on their activity or lifetime. We find different features in the mechanism of selective exposure to pages with respect to the mechanism of selective exposure to topics. In particular, the probability of finding users with high selective exposure to pages increases with the users’ activity, while in the case of topics, selective exposure decreases with activity. However, in both cases, the lifetime of the user has no particular influence on the mechanism of selective exposure.

By comparing the mechanisms of selective exposure to pages and topics, it is possible to differentiate between users’ attention patterns to understand whether they are driven by selective exposure or interest. Our findings suggest that the mechanism of selective exposure, together with users’ limits to attention, strongly affects the way users select and consume news. Further studies and datasets would be needed to investigate whether the presentation priority of the news due to the Facebook algorithm is significantly relevant for the choice of news sources selected and the role that selective exposure plays in the segregation process that leads to the formation of echo chambers.

## Materials and methods

### Topic modeling algorithm

Topic modeling consists in the application of machine learning tools to infer the latent topical structure of a collection of documents.

Well-established and widely used topic models are probabilistic models, such as probabilistic Latent Semantic Analysis (pLSA) [41] and Latent Dirichlet Allocation (LDA) [42], an improvement of pLSA that exploits bayesian statistics), where each document is a mixture of topics while each topic is a mixture of words. Despite being the state of the art method for topic modeling, LDA suffers of several restrictions such as the risk of overfitting and the aprioristic choice of the number of topics [35], among others [43–45]. Such shortcomings of LDA have been recently addressed [35] by exploiting the conceptual relationship between topic modeling and community detection in networks.

By representing the relationship between words and documents (posts in our case) as a bipartite network, the algorithm proposed by [35] detects communities (i.e. cluster of densely interconnected nodes) using a hierarchical Stochastic Block Modeling (hSBM) algorithm [46–48]. The hSBM is a hierarchical version of the stochastic block model (SBM), a generative method for networks with block structure (i.e. communities) that serves as a base for community detection using statistical inference [49, 50].

In [35] a comparison between topic modeling and community detection algorithms, namely pLSA and SBM and LDA and hSBM, is carried on in order to demonstrate the suitability of hSBM for topic modeling problems. In particular, a mixed membership version of the SBM is formally proven to be equivalent to pLSA while the hSBM is shown to be conceptually similar to LDA. In fact, hSBM represents a non parametric bayesian improvement of the SBM in the same way LDA is an improvement of pLSA based on bayesian statistics.

### Data processing and topic modeling

**Fig 6:**
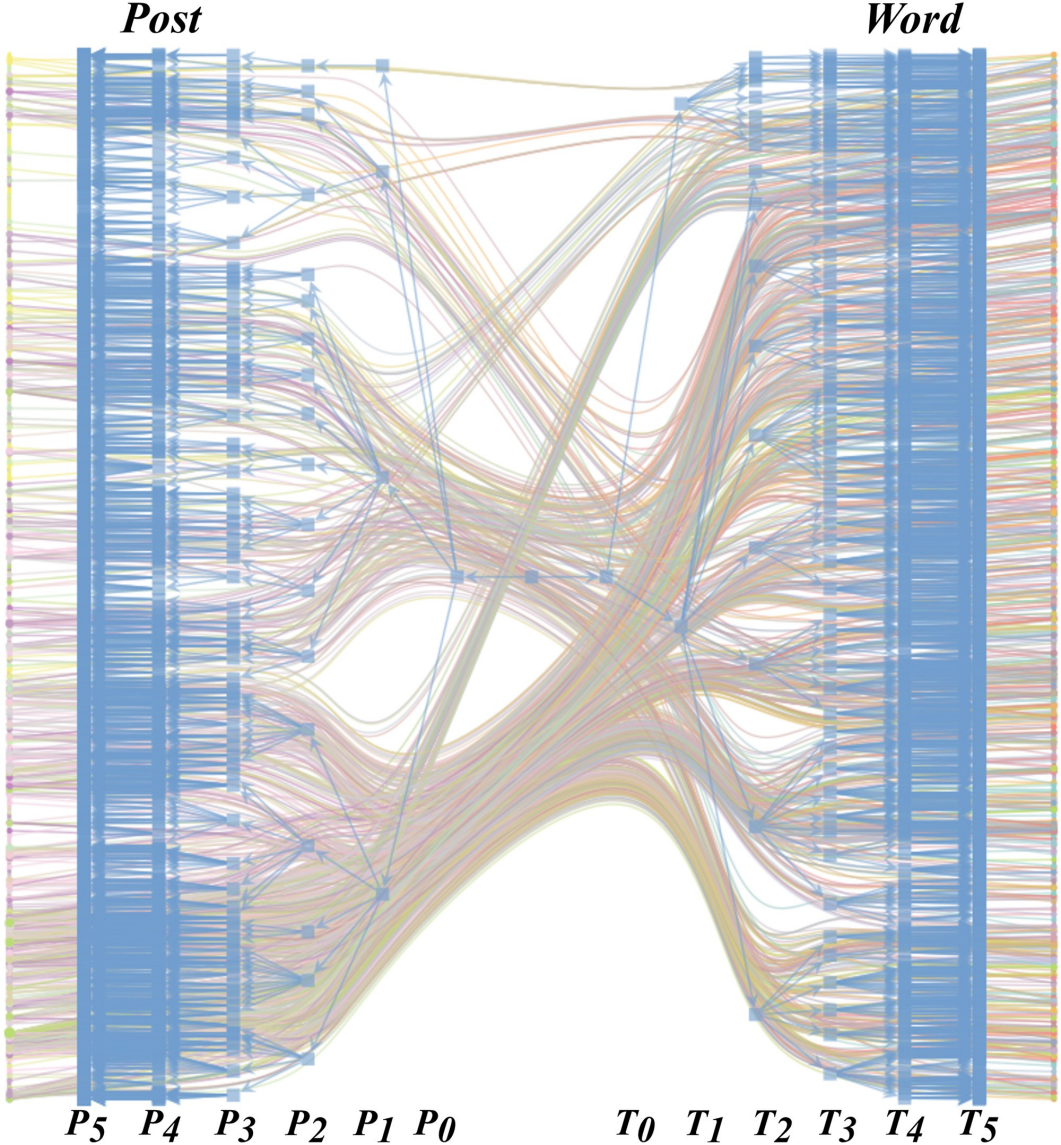
Bipartite representation of the posts-words network. The partition on the left is made up of Facebook posts while the partition on the right is made up of words contained in such posts after pre-processing. The hierarchy obtained from hSBM is reported in blue and has 5 levels.

In our paper we exploit the hSBM algorithm for topic modeling on a bipartite network in which one partition is made up of 50000 pre-processed Facebook posts while the other is made up of the words contained in such posts. The raw Facebook posts that we consider are produced by a set of 583 pages (news outlets). Such posts often include a link to an external website containing an article whose.html file is downloaded, parsed and reported as part of the raw post. The raw posts are then processed in the following way: punctuation and stop words are removed, words are lemmatised, part of speech tagging is executed keeping only nouns, and posts with fewer than five words are removed. After processing the text we run the hSBM algorithm on the considered network. The hSBM splits the bipartite post-word network into groups on different levels organized as a hierarchical tree, as shown in Fig 6. The association between documents and words is represented by the links that underlie the hierarchical tree. Post nodes are displayed on the left side while word nodes are displayed on the right side. On the middle level of the hierarchical tree each node belongs to the same group. On the highest level (*T*_0_ and *P*_0_ respectively) hSBM reflects the bipartite structure into words and document nodes. On each next-higher level the nodes are further divided into word groups (topics) and post groups.

For instance, on the second level of the hierarchy (*T*_2_ and *P*_2_) the hSBM returns 18 topics and 18 clusters of documents. To each document is associated a certain mixture of topics that depends on the words contained in the considered document. Considering the hierarchical tree displayed in Fig 6, in order to investigate selective exposure with respect to topics, we select the level *T*_3_ of the hierarchical tree that contains 91 topics for interpretability reasons. Indeed, we note that such a level displays a good balance between the number of topics (that is not too coarse grained) and their independence from one another We can indeed notice how at lower level of the hierarchy the topics display a somewhat marked overlap that make them hard to to distinguish. Together with these consideration of qualitative nature, we report in Fig 6 of S1 File a comparison between the Gini coefficient with respect to topics considering two levels of the hierarchy and a list of the first five words per each topic. According to the results of the topic modeling algorithm, each topic *t* is present in each post *i* in a certain proportion $p_{t}^{i}\in \left[0,1\right]$ and $\sum_{t=1}^{n_{t}}p_{t}^{i}=1\;\forall i$.

In order to count the number of topics related to each post (as displayed in the inset of Fig 1), we binarize the outcome of the topic modeling as follows. We assume that a post treats a topic if that post is associated to the topic in a certain proportion, determined by the hSBM algorithm, that is greater than zero.

A topic *t* is considered in the pool of topics liked by the user *u* if he/she liked at least one post that contains the topic *t*.

### Gini index

The Gini index can be defined starting from the Gini absolute mean difference Δ [51] of a generic vector *y* with *n* elements, which can be written as:

$$\begin{array}{c} \Delta =\frac{1}{n^{2}}\sum_{i=1}^{n}\sum_{j=1}^{n}\left|y_{i}-y_{j}\right| \end{array}$$ (4)

The relative mean difference is consequently defined as Δ/*μ*_*y*_ where $\mu_{y}=n^{-1}\sum_{i=1}^{n}y_{i}$. Thus, the relative mean difference equals the absolute mean difference divided by the mean of the vector *y*. The Gini index *g* is one-half of the Gini relative mean difference [52]

$$\begin{array}{c} g=\frac{\Delta }{2\mu_{y}} \end{array}$$ (5)

Values of *g* ∼ 1 signal that the considered vector displays high inequality in the distribution of its entries, while values of *g* ∼ 0 signal a tendency towards equality.

### Minimum Gini index

For simplicity, let’s calculate the minimum value the Gini index can attain by considering a user that puts *n*_*likes*_ likes on *n*_*P*_ pages. In this case, our Gini index g* can be written as

$$\begin{array}{c} g^{*}=\frac{1}{2\;n_{P}}\frac{\sum_{P=1}^{n_{P}}\sum_{Q=1}^{n_{P}}\left|I_{uP}^{*}-I_{uQ}^{*}\right|}{\sum_{P=1}^{n_{P}}I_{uP}^{*}} \end{array}$$ (6)

On the one hand, if the user has an overall activity greater than the number of pages, the coefficient $g_{min}^{*}∼0$ since a homogeneous distribution of likes *I*_*uP*_ ∼ *n*_*likes*_/*n*_*P*_ across pages is allowed. On the other hand, when the overall number of likes is smaller than the number of pages, then the minimum value of the Gini index is in general greater than 0, since the likes are concentrated only on *n*_*likes*_ < *n*_*P*_ pages, i.e. the distribution of likes is heterogeneous. Again, in this case we can compute the lower bound $g_{min}^{*}$ to the Gini index, by supposing that the user spreads uniformly his/her likes over *n*_*likes*_ pages (by putting 1 like per page); by substituting in Eq 2, we obtain

$$\begin{array}{c} g_{min}^{*}=\frac{n_{P}-\sum_{P}I_{uP}}{n_{P}} \end{array}$$ (7)

where ∑_*P*_ *I*_*uP*_*n*_*P*_ is the number of likes of the user. Summarising, the coefficient $g_{min}^{*}$ can be written as:

$$\begin{array}{c} g_{min}^{*}=\{\begin{array}{cc} \frac{n_{P}-n_{likes}}{n_{P}} & \text{if}\;n_{likes}\leq n_{P}\\ 0 & \text{otherwise}\; \end{array} \end{array}$$ (8)

### Data collection

The Europe Media Monitor provides a list of all news sources. We limit our collection to Facebook pages associated to such sources reporting in English. The downloaded data from each page include all of the posts made from 1 January 2010 to 31 December 2015, as well as all of the likes and comments on those posts. In this paper we consider a sample of the original dataset made up of 50000 posts produced by 583 pages spanning the six-year time window.

### Ethics statement

The entire data collection process is performed exclusively by means of the Facebook Graph API which is publicly available (under the following limitations https://developers.facebook.com/docs/graph-api/reference/v6.0/page/feed) and can be used through one’s personal Facebook user account. We used only publicly available data. Users with privacy restrictions are not included in our dataset. Data is downloaded from Facebook pages that are public entities. When allowed by users’ privacy specifications, we access public information. However, in this project we use fully anonymized and aggregated data. We abide by the terms, conditions, and privacy policies of Facebook. Due to an update to Facebook API policy on Feb 5 2018, we are not allowed to access any information about the users who reacted/commented to Facebook content on public pages. Such information is available only to the Facebook page owners. The list of pages is available at: github.com/cinhelli/SelectiveExposureFBNewsDiet.

## Supporting information

S1 File (PDF)

S1 Data (HTML)
