# Supplementary material for: Selective exposure shapes the Facebook news diet
Source: PLoS One. 2020 Mar 13;15(3):e0229129. doi: 10.1371/journal.pone.0229129 (PMC7069632; doi:10.1371/journal.pone.0229129)
Supplement: S1 File — (PDF) [file pone.0229129.s001.pdf]

Selective Exposure shapes the Facebook News Diet

Matteo Cinelli,[1,*] Emanuele Brugnoli,[1] Ana Lucia Schmidt,[2]
Fabiana Zollo,[2,1] Walter Quattrociocchi,[2,1] and Antonio Scala[1,3]
[1]*Applico Lab, CNR-ISC*
[2]*Università di Venezia "Ca' Foscari"*
[3]*LIMS, the London Institute for Mathematical Sciences*

SUPPLEMENTARY INFORMATION

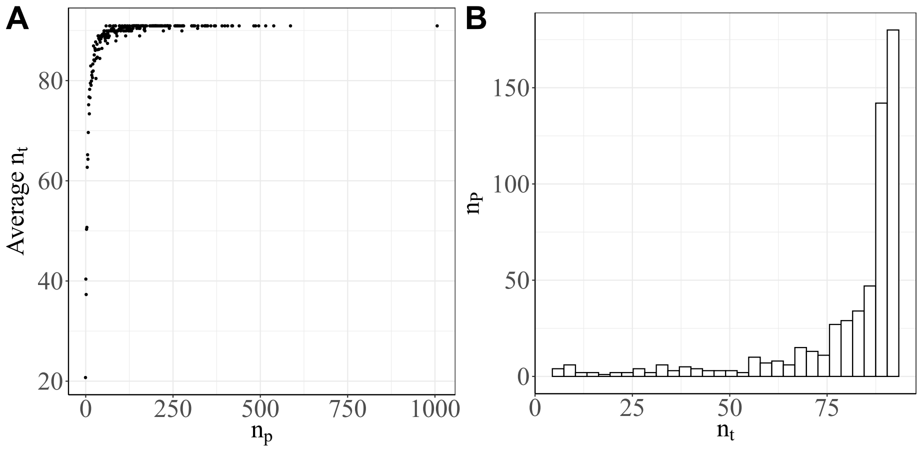

FIG. 1. **Number of posts and number of topics covered by pages.** Left panel: Average number of topics covered by a page, given its activity (measured as number of posts). We observe that the average number of topics reaches a plateau of ~ 90 topics for pages with an activity of more than ~ 30 posts. Right panel: Number of pages that cover a certain number of topics. We observe that most of the pages tend to cover most of the of topics.

* matteo.cinelli@roma1.infn.it

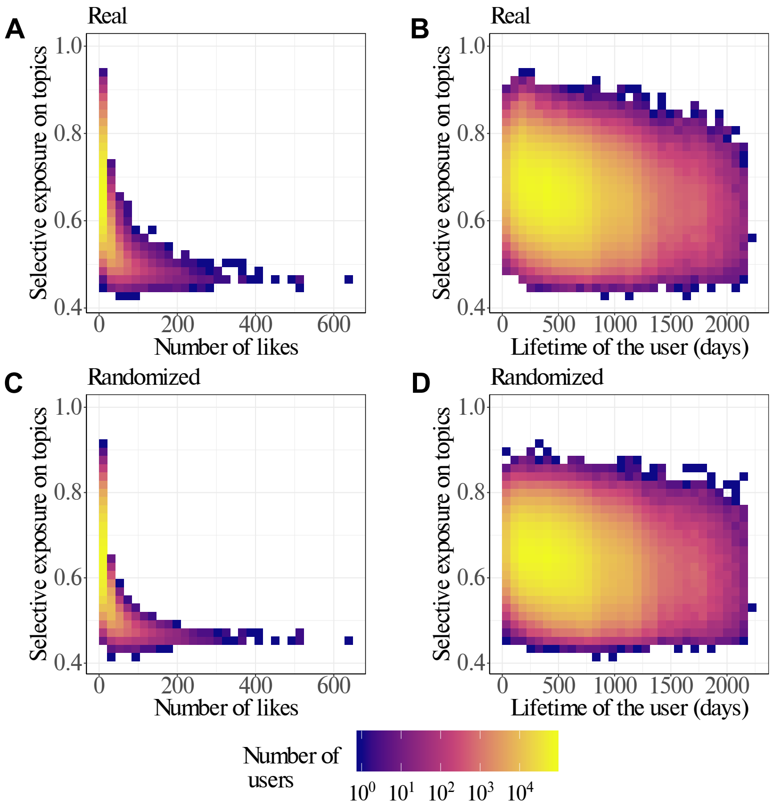

FIG. 2. **Correlation among users selective exposure to topics (as measured by the Gini coefficient $g^\dagger$) and their activity/lifetime.** Data are pre-processed in order to keep only users with at least 5 likes. The colour scale of the distributions represents the number of users related to a certain (x, y) couple. Results of the randomization of the actual data are reported in the bottom panels. Panel A: the distribution of selective exposure to topics with respect to users activity shows that increasing activity levels correspond to lower selective exposure, i.e. users concentrate on a higher number of topics. Panel B: the distribution of selective exposure to topics with respect to users lifetime (measured in days) shows that the mechanism of choice of topics does not seem to be influenced by the time users have been present on the social medium. Panels C and D: the distribution of selective exposure can be replicated after randomizing the user's liking pattern.

In order to shed light on the issue of topical selectivity of users we computed the evolution of the Gini coefficient for the *maximally selective* user, i.e. for the user who likes only posts that are focused on a specific topic. The procedure used to compute the selective exposure of such a user is reported, by means of an example, in the blocks of Table 1. The main idea behind this procedure is to identify posts that are very topic-specific and to use them in order to compute the Gini coefficient of the maximally selective user. The mixture of topics of each post is not synthetic but it is obtained using the real mixtures deriving from the topic modeling algorithm applied on

the analyzed posts. For instance, supposing that the number of topics is 4 (while it is 91) a possible result of the mixed membership model could be that represented in the first block (four rows) of Table 1. Given the results of the mixed membership, the concentration of posts is computed using the Localization parameter. The parameter is defined as $L[\phi] = \frac{\sum_i (\phi_i^2)^2}{\sum_i \phi_i^4}$ where ϕ_i is the proportion of the topic i treated by a certain post. Thus, in the case in which a post only treats one topic, $L = 1$. Conversely, if a post treats equally all the n_t topics (i.e. $\phi_i = 1/n_t$) then $L = n_t$. The example values of the Localization coefficient are reported in Table 1. The most localized posts are those treating only one topic (i.e. b and d) while post a is less localized than post c.

We assumed that the number of likes of the user ranges from 1 to the maximum number of likes that is 633, in order to span all the possible ranges of activity. Similarly to the example, the user who is very selective towards a certain topic is obtained by choosing the first 633 posts with the lowest localization coefficient (as shown in the second block of Table 1) and sorting the membership of such posts to topics in non-increasing order (as shown in the third block of Table 1). In such a way, the first topic is the topic most treated by the posts used for the computation of the Gini coefficient and from such a membership we can simulate the choice of the maximally selective user while respecting the proportions (not the order though) of topics within posts. The average localization coefficient of the 633 posts that we take into account in real data is $\overline{L} = 1.125$.

In Figure 3 of this document, we plot the evolution of the Gini coefficient of the maximally selective user (dashed line) according to the procedure that we have explained. The maximally selective user is indeed very selective since it displays high values of the Gini coefficient ($G_{max} = 1$ and $G_{min} = 0.87$) that, even if decreasing, seem to converge to a steady state with the increase of activity. The evolution of the Gini coefficient for the maximally selective user is far from the empirical distribution of the Gini coefficient, yet it follows a similar trend, thus implying a certain departure of real users from maximal topical selectivity.

**initial topic membership**					
post/topic	1	2	3	4	Localization
a	0.2	0	0.8	0	1.125
b	1	0	0	0	1
c	0	0.9	0	0.1	1.025
d	0	0	0	1	1
**sort by Localization**					
b	1	0	0	0	1
d	0	0	0	1	1
c	0	0.9	0	0.1	1.025
a	0.2	0	0.8	0	1.125
**reassign topic membership**					
b'	1	0	0	0	1
d'	1	0	0	0	1
c'	0.9	0.1	0	0	1.025
a'	0.8	0.2	0	0	1.125

TABLE I. Procedure used to determine selective exposure of the maximally selective user.

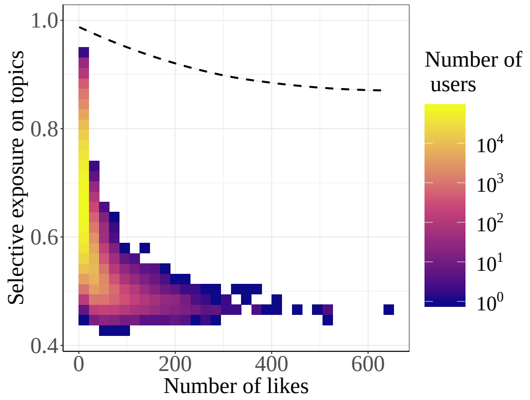

FIG. 3. **Model of a user displaying maximal topical selectivity.** The evolution of the Gini index of the selective user is represented as a dashed line. In the background we also report the correlation between users selective exposure to topics (as measured by the Gini coefficient $g^{\dagger}$) and their activity.

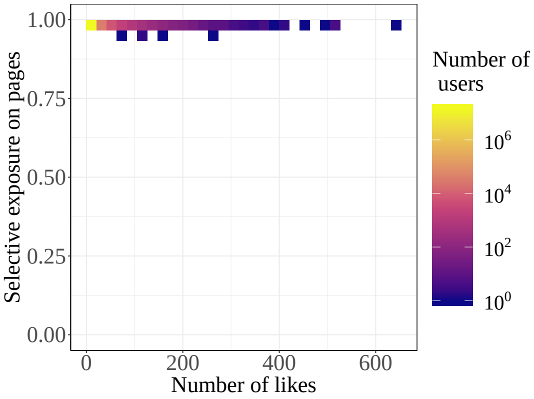

FIG. 4. **Selective exposure to pages measured using g^*, the Gini index without correction.** The colour scale of the distribution represents the number of users related to a certain (x, y) couple.

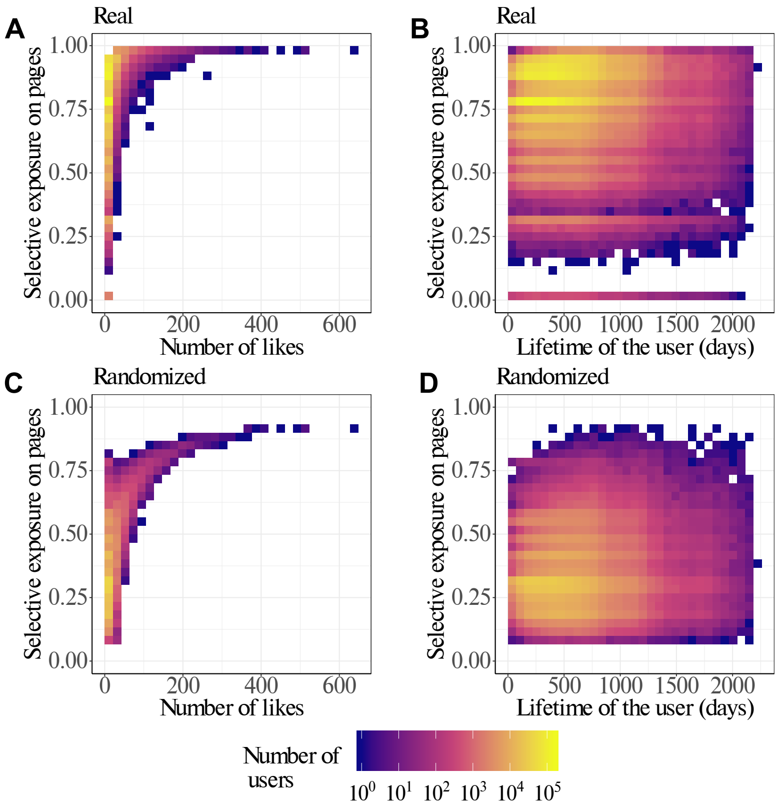

FIG. 5. **Correlation among users' selective exposure (as measured by the Gini coefficient $g^{\triangleright}$) and their activity/lifetime.** Data are pre-processed in order to keep only users with at least 5 likes. The colour scale of the distributions represents the number of users related to a certain (x, y) couple. Results of the randomization of the actual data are reported in the bottom panels. Panel A: the distribution of selective exposure to pages with respect to users' activity shows that increasing activity levels correspond to higher selective exposure, i.e. users concentrate on fewer pages. Panel B: the distribution of selective exposure to pages with respect to users' lifetime (measured in days) shows that the mechanism of choice of pages does not seem to be influenced by the time users have been present on the social medium. The results of the panels are consistent with a way of choosing news outlets based on selective exposure rather than on a comparison among several sources; it is also consistent with a reinforcement mechanism for which the higher the activity, the stronger the concentration on fewer pages. Panels C and D: extreme values of selective exposure, observed in the actual data, cannot be reached after the randomization process.

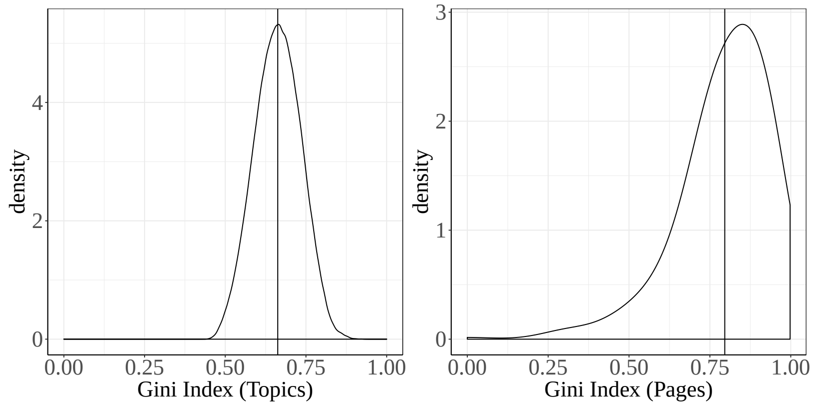

FIG. 6. **Probability density function of selective exposure.** Left panel: the Gini index $g^{\dagger}$ displays a Gaussian distribution centered over the mean. Right panel: the Gini index $g^{\triangleright}$ displays a Gaussian-like distribution with right skewness. Such skewness explains the light shift of the mean value from the peak of the distribution.

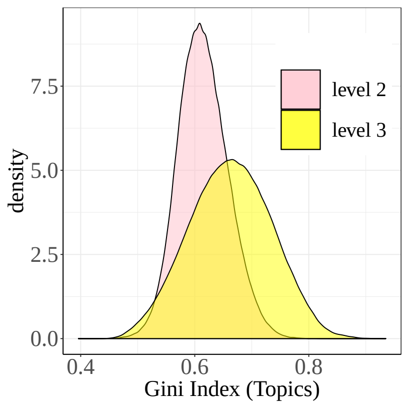

FIG. 7. **Distribution of the Gini index for two levels of the hSBM**. Level 2 (18 topics) and level 3 (91 topics) of the hierarchy provided by the hSBM algorithm both diplay a Gaussian distribution of the Gini index. In the case of higher number of topics (level 3) the Gini index spans a larger interval meaning that choosing such a level for topic modeling allows a higher degree of diversity in the interaction with topics than choosing level 2.

TABLE II. **First five words per topic in the case $n_t = 91$ - 1/3**

	Topic 1	Topic 2	Topic 3	Topic 4	Topic 5	Topic 6
1	time	country	dublin	police	attempt	news
2	people	government	wale	court	speech	life
3	day	support	rugby	crime	status	family
4	world	law	cork	victim	session	woman
5	generosity	nation	assumption	justice	labour	child
	Topic 7	Topic 8	Topic 9	Topic 10	Topic 11	Topic 12
1	medium	center	facebook	town	party	source
2	account	program	list	yesterday	election	december
3	network	washington	history	october	campaign	thousand
4	contact	limit	picture	station	vote	strike
5	voice	speed	book	train	politic	pressure
	Topic 13	Topic 14	Topic 15	Topic 16	Topic 17	Topic 18
1	story	month	peace	risk	syria	comment
2	photo	report	freedom	study	saudi	term
3	break	call	army	research	turkey	issue
4	night	head	weapon	space	iraq	information
5	morning	view	terrorist	safety	arabia	community
	Topic 19	Topic 20	Topic 21	Topic 22	Topic 23	Topic 24
1	food	friend	health	attack	subscriber	team
2	build	heart	care	force	premium	update
3	land	injury	girl	war	till	app
4	water	tomorrow	live	fight	johnston	phone
5	travel	ticket	parent	threat	apologise	camera
	Topic 25	Topic 26	Topic 27	Topic 28	Topic 29	Topic 30
1	respect	death	event	hour	week	site
2	church	kill	newsletter	street	city	experience
3	blame	hospital	analysis	air	plan	owner
4	religion	incident	web	fire	deal	date
5	belief	spokesman	coverage	staff	job	offer
	Topic 31	Topic 32	Topic 33	Topic 34	Topic 35	Topic 36
1	navigation	scene	economy	video	house	offensive
2	burn	celebrity	trade	twitter	director	telegraph
3	whale	steal	energy	tweet	university	syndication
4	dolphin	tribute	oil	aria	student	accessibility
5	dig	cop	gas	lucan	education	indulge

TABLE III. **First five words per topic in the case $n_t = 91$ - 2/3**

	Topic 37	Topic 38	Topic 39	Topic 40	Topic 41	Topic 42
1	share	park	series	trial	music	subscribe
2	november	toronto	london	gun	film	edition
3		ride	couple	prosecutor	style	inbox
4		texa	reveal	sentence	movie	upgrade
5		chicago	spot	penalty	entertainment	columnist
	**Topic 43**	**Topic 44**	**Topic 45**	**Topic 46**	**Topic 47**	**Topic 48**
1	senate	security	minister	service	newspaper	island
2	mayor	chief	leader	business	press	foot
3	barack	african	power	company	shoot	sea
4	senator	union	secretary	money	officer	coast
5	immigration	association	commission	bank	charge	ship
	**Topic 49**	**Topic 50**	**Topic 51**	**Topic 52**	**Topic 53**	**Topic 54**
1	technology	degree	map	percent	policy	york
2	datum	sound	consent	worker	website	california
3	feature	tree	password	tax	email	encourage
4	user	sign	malta	budget	article	advance
5	review	cat	instruction	finance	content	llc
	**Topic 55**	**Topic 56**	**Topic 57**	**Topic 58**	**Topic 59**	**Topic 60**
1	market	minute	fee	background	road	department
2	rate	player	cancer	engage	car	measure
3	industry	field	professor	jamaican	traffic	practice
4	increase	goal	weight	extension	vehicle	addition
5	board	box	cell	adhere	driver	protection
	**Topic 61**	**Topic 62**	**Topic 63**	**Topic 64**	**Topic 65**	**Topic 66**
1	pic	sport	cyber	price	american	russia
2	cape	fan	coup	sale	organization	ukraine
3	blow	stage	thailand	product	trend	moscow
4	legend	football	hacker	shop	reuter	sanction
5	advocate	race	bangkok	store	quote	putin
	**Topic 67**	**Topic 68**	**Topic 69**	**Topic 70**	**Topic 71**	**Topic 72**
1	radio	county	document	net	japan	philippine
2	trump	globe	text	apple	korea	cbn
3	flag	boston	location	fake	beijing	manila
4	dec	lake	window	brexit	taiwant	usadvertise
5	shape	landmark	display	tablet	tokyo	ang

TABLE IV. **First five words per topic in the case $n_t = 91$ - 3/3**

	Topic 73	Topic 74	Topic 75	Topic 76	Topic 77	Topic 78
1	australia	character	reader	gold	detail	game
2	singapore	icon	editor	package	contribution	bowl
3	sir	archive	mail	metal	labor	fox
4	panic	linkedin	gift	closure	arrival	basketball
5	tobago	dispatch	donation	silver	gender	giant
	**Topic 79**	**Topic 80**	**Topic 81**	**Topic 82**	**Topic 83**	**Topic 84**
1	nigeria	europe	hit	development	con	pakistan
2	ghana	summit	hunt	region	dive	khan
3	trademark	enterprise	earthquake	agreement	persona	delhi
4	nollywood	greece	magnitude	resource	sul	islamabad
5	gist	brussel	quake	population	tempo	wisdom
	**Topic 85**	**Topic 86**	**Topic 87**	**Topic 88**	**Topic 89**	**Topic 90**
1	alert	read	advertisement	title	nairobi	mexico
2	gallery	language	malaysia	method	bulawayo	brazil
3	category	button	rss	commentary	hostage	colombia
4	salary	banner	colour	venue	somalia	rio
5	notification	description	malaysiakini	symbol	chave	chile
	**Topic 91**					
1	migration					
2	adapt					
3	flat					
4	namibia					
5	madam					

TABLE V. **First five words per topic in the case** $n_t = 18$

	Topic 1	Topic 2	Topic 3	Topic 4	Topic 5	Topic 6
1	news	country	team	police	development	death
2	time	government	game	policy	region	newspaper
3	people	month	sport	house	food	press
4	day	report	update	minister	department	shoot
5	world	comment	app	party	price	officer
	**Topic 7**	**Topic 8**	**Topic 9**	**Topic 10**	**Topic 11**	**Topic 12**
1	story	event	subscriber	detail	character	video
2	photo	newsletter	premium	radio	icon	twitter
3	break	analysis	advertisement	trial	archive	subscribe
4	night	web	map	respect	hit	edition
5	hour	syria	consent	senate	cyber	inbox
	**Topic 13**	**Topic 14**	**Topic 15**	**Topic 16**	**Topic 17**	**Topic 18**
1	title	share	reader	nigeria	europe	con
2	read	november	editor	ghana	american	migration
3	language	philippine	county	trademark	organization	dive
4	gold	mexico	fee	nollywood	russia	persona
5	package	cbn	mail	gist	trend	sul
