# Supplementary material for: Selective exposure shapes the Facebook news diet
Source: PLoS One. 2020 Mar 13;15(3):e0229129. doi: 10.1371/journal.pone.0229129 (PMC7069632; doi:10.1371/journal.pone.0229129)
Supplement: S1 Data — (HTML) [file pone.0229129.s002.html]

|  | Name | Website | Facebook ID | Country | Region |
| --- | --- | --- | --- | --- | --- |
| 1 | 24 Tanzania | http://24tanzania.com/ | 142633892541250 | Tanzania | Africa |
| 2 | 680 News | http://www.680news.com/ | 204410527704 | USA | North America |
| 3 | 7 Days UAE | http://7days.ae/ | 134378466585520 | United Arab Emirates | Middle East |
| 4 | 7 News Belize | http://www.7newsbelize.com/ | 523592704341166 | Belize | North America |
| 5 | +972 Magazine | http://972mag.com/ | 148081438555256 | Israel | Middle East |
| 6 | 9 News Australia | http://www.9news.com.au/ | 107637365950776 | Australia | Oceania |
| 7 | Australian Broadcasting Corporation | http://www.abc.net.au/ | 194764094549 | Australia | Oceania |
| 8 | abc7NY | http://7online.com/ | 31160214090 | USA | North America |
| 9 | ABC News | http://abcnews.go.com/ | 86680728811 | USA | North America |
| 10 | ABS-CBN News | http://www.abs-cbnnews.com/ | 27254475167 | Philippines | Asia |
| 11 | ABS TV/Radio | http://abstvradio.com/ | 558698930896002 | Antigua and Barbuda | Central America |
| 12 | AFP News Agency | http://www.afp.com/en | 155857464452265 | France | European Union |
| 13 | Africa Intelligence | http://www.africaintelligence.com/ | 1600441596851782 | France | European Union |
| 14 | African Christian Democratic Party | http://www.acdp.org.za/ | 379138726819 | South Africa | Africa |
| 15 | African Brains | http://africanbrains.net/ | 137238649646766 | South Africa | Africa |
| 16 | African Business Magazine | http://www.africanbusinessmagazine.com/ | 114117578656259 | United Kingdom | European Union |
| 17 | African Mining | http://www.africanmining.com/ | 413345415394061 | South Africa | Africa |
| 18 | African Review | http://www.africanreview.com/ | 507239115959583 | United Kingdom | European Union |
| 19 | Ahram Online | http://english.ahram.org.eg/ | 138561829527411 | Egypt | Africa |
| 20 | Airforce Tecnology | http://www.airforce-technology.com | 376588539031515 | United Kingdom | European Union |
| 21 | Air Transport World | http://atwonline.com/ | 98452961409 | USA | North America |
| 22 | The Atlanta Journal-Constitution | http://www.ajc.com/ | 13310147298 | USA | North America |
| 23 | AJW - Asia & Japan Watch | http://ajw.asahi.com/ | 157466287640348 | Japan | Asia |
| 24 | Al-Ahram Weekly | http://weekly.ahram.org.eg/Index.aspx | 444452792278401 | Egypt | Africa |
| 25 | Al Bawaba News | http://www.albawaba.com/en/ | 145270758819050 | Jordan | Middle East |
| 26 | Al Jazeera | http://english.aljazeera.net/ | 7382473689 | Qatar | Middle East |
| 27 | AllAfrica | http://allafrica.com | 98946450029 | USA | North America |
| 28 | All Ghana News | http://www.allghananews.com/ | 110299945719263 | Ghana | Africa |
| 29 | Almal News | http://en.almalnews.com/ | 275916392585253 | Egypt | Africa |
| 30 | Al Manar | http://www.almanar.com.lb/english/main.php | 567297210065647 | Lebanon | Middle East |
| 31 | AlterNet | http://www.alternet.org/ | 17108852506 | USA | North America |
| 32 | Amandala Newspaper | http://amandala.com.bz/news/ | 100548070049565 | Belize | North America |
| 33 | Ambergris Today | http://www.ambergristoday.com/ | 150068098371942 | Belize | North America |
| 34 | Center for American Progress | https://www.americanprogress.org/ | 6072343558 | USA | North America |
| 35 | Ammon News - English | http://en.ammonnews.net/ | 124114467624068 | Jordan | Middle East |
| 36 | Amnesty International | https://www.amnesty.org/en/ | 111658128847068 | United Kingdom | European Union |
| 37 | Athens News Agency - Macedonian Press Agency | http://www.amna.gr/english/ | 324281057673335 | Greece | European Union |
| 38 | Anadolu Agency - English | http://www.aa.com.tr/en | 1469323633339182 | Turkey | EU Candidate |
| 39 | An Garda S�och�na | http://www.garda.ie/ | 167613868000 | Ireland | European Union |
| 40 | The Anglo-Celt | http://www.anglocelt.ie/ | 125313887068 | Ireland | European Union |
| 41 | Associated Press | http://www.ap.org/ | 249655421622 | USA | North America |
| 42 | Arab Times | http://www.arabtimesonline.com/ | 185411171491289 | Kuwait | Middle East |
| 43 | Oratert | http://www.oratert.com/news/ | 219526301441279 | Armenia | Asia |
| 44 | Army Technology | http://www.army-technology.com | 273161589364941 | USA | North America |
| 45 | ArtMatters.Info | http://artmatters.info/ | 156915596222 | Kenya | Africa |
| 46 | Asbarez | http://www.asbarez.com/ | 30695878200 | Armenia | Asia |
| 47 | Americas Society - Council of the Americas | http://www.as-coa.org/ | 22309058308 | USA | North America |
| 48 | Asharq Al-Awsat English Edition | http://www.aawsat.net/ | 135349813159197 | Lebanon | Middle East |
| 49 | Asia News Network | http://www.asianewsnet.net/home/ | 291155398585 | Thailand | Asia |
| 50 | Asian Tribune | http://www.asiantribune.com/ | 85012674846 | Thailand | Asia |
| 51 | Asia Times | http://www.atimes.com/ | 186423081422922 | China | Asia |
| 52 | Australia Network News | http://australianetworknews.com/ | 1569497949997753 | Australia | Oceania |
| 53 | Aviation Week | http://aviationweek.com/ | 16067432199 | USA | North America |
| 54 | Awate | http://awate.com/ | 211930818821450 | Eritrea | Africa |
| 55 | The Arizona Republic | http://www.azcentral.com/ | 50978409031 | USA | North America |
| 56 | AzerNews | http://www.azernews.az/ | 138411436293144 | Azerbaijan | Asia |
| 57 | Azo Mining | http://www.azomining.com/ | 195005930530874 | United Kingdom | European Union |
| 58 | B92 English | http://www.b92.net/eng/ | 294076883992251 | Serbia | European Other |
| 59 | Bakhtar News Agency | http://www.bakhtarnews.com.af/eng/ | 128677440577060 | Afghanistan | Middle East |
| 60 | Balkan Insight | http://www.balkaninsight.com/en/page/all-balkans-home | 97956845505 | United Kingdom | European Union |
| 61 | The Baltimora sun | http://www.baltimoresun.com/ | 9299179711 | USA | North America |
| 62 | Bangkok Post | http://www.bangkokpost.com/ | 133643127712 | Thailand | Asia |
| 63 | Banknet India | http://www.banknetindia.com/ | 156945944397481 | India | Asia |
| 64 | Barents Observer | http://www.barentsobserver.com/ | 196717396635 | Norway | European Other |
| 65 | Barron's | http://www.barrons.com/ | 64579042740 | USA | North America |
| 66 | BBC iPlayer Radio | http://www.bbc.co.uk/radio/ | 1470145583204829 | United Kingdom | European Union |
| 67 | BBC News | http://www.bbc.com/news/ | 228735667216 | United Kingdom | European Union |
| 68 | Beat 102-103 | http://www.beat102103.com/ | 117022648342887 | Ireland | European Union |
| 69 | BelTA - Belarusian Telegraph Agency | http://eng.belta.by/ | 159485357443987 | Belarus | European Other |
| 70 | News Letter | http://www.newsletter.co.uk/ | 117370764948881 | United Kingdom | European Union |
| 71 | Belfast Telegraph | http://www.belfasttelegraph.co.uk/ | 237692023818 | United Kingdom | European Union |
| 72 | The Bermuda Sun | http://www.bermudasun.org/ | 12844875188 | Bermuda | North America |
| 73 | BetaNews | http://betanews.com/ | 167777169963870 | USA | North America |
| 74 | Better Diamond Initiative | http://betterdiamondinitiative.org/ | 474528435937430 | USA | North America |
| 75 | BioFuels Journal | http://www.biofuelsjournal.com/ | 785884781480346 | USA | North America |
| 76 | Beijing Review | http://www.bjreview.com.cn/ | 164344500263690 | China | Asia |
| 77 | Bloomberg News | http://www.bloomberg.com/ | 266790296879 | USA | North America |
| 78 | Bahrain News Agency | http://www.bna.bh/portal/en | 155290664516690 | Bahrain | Middle East |
| 79 | BNO News | http://bnonews.com/news/index.php/news/landing | 60109657413 | USA | North America |
| 80 | Boston | http://www.boston.com/ | 6879409364 | USA | North America |
| 81 | Boston Haitian Reporter | http://www.bostonhaitian.com/ | 132342263485363 | Haiti | Central America |
| 82 | Boston Herald | http://bostonherald.com/ | 197211981599 | USA | North America |
| 83 | Botswana Guardian | http://www.botswanaguardian.co.bw/ | 261592817198239 | Botswana | Africa |
| 84 | Breaking News | http://www.breakingnews.ie | 120689931275023 | Ireland | European Union |
| 85 | The Argus | http://www.theargus.co.uk/ | 57197526698 | United Kingdom | European Union |
| 86 | Brisbane Times | http://www.brisbanetimes.com.au/ | 95683517460 | Australia | Oceania |
| 87 | The Brookings Institution | http://www.brookings.edu/ | 137459917707 | Colombia | South America |
| 88 | The Broad Street Journal | http://www.broadstreetjournalbarbados.com/ | 306701519754 | Barbados | Central America |
| 89 | Budapest Business Journal | http://www.bbj.hu/ | 162210567199344 | Hungary | European Union |
| 90 | The Budapest Times | http://budapesttimes.hu/ | 473754729371730 | Hungary | European Union |
| 91 | Buenos Aires Herald | http://www.buenosairesherald.com/ | 333788759997981 | Argentina | South America |
| 92 | Bulatlat | http://www.bulatlat.com/ | 63467907915 | Philippines | Asia |
| 93 | Bulawayo 24 News | http://www.bulawayo24.com/ | 128990327163597 | Zimbabwe | Africa |
| 94 | Bulgarian Telegraph Agency | http://bta.bg/en | 649620041801768 | Bulgaria | European Union |
| 95 | Business Daily Africa | http://www.businessdailyafrica.com/ | 111397575568693 | Kenya | Africa |
| 96 | Business Day Live | http://www.businessday.co.za/ | 286638794693284 | South Africa | Africa |
| 97 | Business & Human Rights Resource Centre | http://business-humanrights.org/en | 10510773883 | Ireland | European Union |
| 98 | Business Wire | http://www.businesswire.com/ | 82442677571 | USA | North America |
| 99 | BusinessWorld Online | http://www.bworldonline.com/ | 115158345163134 | Philippines | Asia |
| 100 | Republic of Botswana | http://www.gov.bw/ | 148228411926492 | Botswana | Africa |
| 101 | The Belize Times | http://www.belizetimes.bz/ | 325514113131 | Belize | North America |
| 102 | Caf�babel | http://www.cafebabel.co.uk/ | 357343795001 | United Kingdom | European Union |
| 103 | The Cameroon Daily Journal | http://www.cameroonjournal.com/ | 1497726193822330 | Cameroon | Africa |
| 104 | Cameroon Online | http://www.cameroononline.org/ | 287988637383 | Cameroon | Africa |
| 105 | AKIpress Central Asian News Service | http://en.ca-news.org/ | 138634076266590 | Kyrgyzstan | Middle East |
| 106 | Caperi | http://www.capitaleritrea.com/ | 257893353570 | Eritrea | Africa |
| 107 | Cape Verde | http://www.capeverde.com/ | 328585463869159 | Cape Verde | Africa |
| 108 | Capital FM | http://www.capitalfm.co.ke/ | 178342827608 | Kenya | Africa |
| 109 | CARE | http://www.care.org/ | 30139072158 | USA | North America |
| 110 | Caribbean360 | http://www.caribbean360.com/ | 147441385288431 | Barbados | Central America |
| 111 | Carlow Nationalist | http://www.carlow-nationalist.ie/ | 233149060080354 | Ireland | European Union |
| 112 | Carnegie Endowment for International Peace | http://carnegieendowment.org/ | 62935143720 | USA | North America |
| 113 | Carnegie Europe | http://carnegieeurope.eu/ | 74341131538 | Belgium | European Union |
| 114 | Carnegie Moscow Center | http://carnegie.ru/?lang=en | 111281202235293 | Russia | Asia |
| 115 | Cato Institute | http://www.cato.org/ | 26668999076 | USA | North America |
| 116 | Caucasian Knot | http://www.eng.kavkaz-uzel.ru/ | 129869803756134 | Russia | Asia |
| 117 | Cayman Compass | http://www.caycompass.com/ | 237869980023 | Cayman Islands | North America |
| 118 | CBC News | http://www.cbc.ca | 5823419603 | Canada | North America |
| 119 | Caribbean Broadcasting Corporation | http://www.cbc.bb/index.php/en/ | 1447053148908724 | Barbados | Central America |
| 120 | CBS Baltimore | http://baltimore.cbslocal.com/ | 261323008476 | USA | North America |
| 121 | CBS News | http://www.cbsnews.com | 131459315949 | USA | North America |
| 122 | CBS Radio | http://www.cbsradio.com/ | 165760583475583 | USA | North America |
| 123 | CCN TV6 | http://www.tv6tnt.com/ | 192696260772574 | Trinidad and Tobago | South America |
| 124 | Center on Budget and Policy Priorities | http://www.cbpp.org/ | 42002969352 | USA | North America |
| 125 | Centre for Asia Pacific Aviation | http://www.centreforaviation.com/ | 212338015492650 | United Kingdom | European Union |
| 126 | CERN | http://home.web.cern.ch/ | 169005736520113 | Switzerland | European Other |
| 127 | CFO | http://www.cfo.com/ | 146601585370995 | USA | North America |
| 128 | Channel 4 News | http://www.channel4.com/news/ | 6622931938 | United Kingdom | European Union |
| 129 | Channel Africa | http://www.channelafrica.org/portal/site/ChannelAfrica/ | 287474612524 | South Africa | Africa |
| 130 | Channel NewsAsia | http://www.channelnewsasia.com | 93889432933 | Singapore | Asia |
| 131 | Chatham House | http://www.chathamhouse.org/ | 202798122688 | United Kingdom | European Union |
| 132 | Chicago Tribune | http://www.chicagotribune.com/ | 5953023255 | USA | North America |
| 133 | China.com | http://english.china.com/ | 623480657770927 | China | Asia |
| 134 | China Daily | http://www.chinadaily.com.cn | 191347651290 | China | Asia |
| 135 | China.org.cn | http://www.china.org.cn | 371171589575669 | China | Global |
| 136 | China Plus News - CRI | http://english.cri.cn/ | 223495844457800 | China | Asia |
| 137 | The China Post | http://www.chinapost.com.tw/ | 143632722314869 | China | Asia |
| 138 | The Christian Science Monitor | http://www.csmonitor.com/ | 14660729657 | USA | North America |
| 139 | The Houston Chronicle | http://www.chron.com/ | 12852567813 | USA | North America |
| 140 | CIP Americas Program | http://www.cipamericas.org/ | 113343085368711 | USA | North America |
| 141 | City AM | http://www.cityam.com/ | 213682385348579 | United Kingdom | European Union |
| 142 | Civil.ge | http://www.civil.ge/eng/ | 154006094635924 | Georgia | Asia |
| 143 | Clare FM | http://www.clare.fm | 76448532790 | Ireland | European Union |
| 144 | CNBC | http://www.cnbc.com/ | 97212224368 | USA | North America |
| 145 | CNC3 | http://www.cnc3.co.tt/ | 126206357995 | Trinidad and Tobago | South America |
| 146 | CNET News | http://news.cnet.com/ | 7155422274 | USA | North America |
| 147 | CTV | http://www.ctntworld.com/cnews2/index.php | 377693645609 | Trinidad and Tobago | South America |
| 148 | CNN International | http://edition.cnn.com/ | 18793419640 | USA | North America |
| 149 | CNNMoney news | http://money.cnn.com/ | 6651543066 | USA | North America |
| 150 | Colombia Reports | http://colombiareports.co/ | 64680372407 | Colombia | South America |
| 151 | European Union - Committee of the Regions | http://cor.europa.eu/en/Pages/home.aspx | 527351247377965 | Belgium | European Union |
| 152 | Common Ground News Service | http://www.commongroundnews.org/index.php | 60149902595 | USA | North America |
| 153 | Congress Rental Network | http://congressrentalnetwork.com/ | 554092937948891 | Malta | European Union |
| 154 | The Connaught Telegraph | http://www.con-telegraph.ie/ | 187794613261 | Ireland | European Union |
| 155 | Construction Week Online | http://www.constructionweekonline.com/ | 150848527337 | USA | North America |
| 156 | The Copenhagen Post | http://cphpost.dk/ | 38409311585 | Denmark | European Union |
| 157 | Cork's 96FM | http://www.96fm.ie/ | 194278177271109 | Ireland | European Union |
| 158 | Cornwall Seaway News | http://www.cornwallseawaynews.com/ | 120383281376364 | Canada | North America |
| 159 | Courier Journal | http://www.courier-journal.com/ | 69357466992 | CostaRica | Central America |
| 160 | The Courier Mail | http://www.couriermail.com.au/ | 90004797701 | Australia | Oceania |
| 161 | CPAC - Cable Public Affairs Channel | http://www.cpac.ca/en/ | 8260742627 | Canada | North America |
| 162 | CPI Financial | http://www.cpifinancial.net/news | 509446489112881 | USA | North America |
| 163 | International Crisis Group | http://www.crisisgroup.org/ | 341675908125 | Belgium | European Union |
| 164 | The Critical Threats Project | http://www.criticalthreats.org/ | 131736233536920 | USA | North America |
| 165 | CSIS - Center for Strategic and International Studies | http://csis.org/ | 118399079197 | USA | North America |
| 166 | CTV News | http://www.ctvnews.ca/ | 194553860586548 | Canada | North America |
| 167 | Cyprus Expat | http://www.cyprusexpat.co.uk/ | 357342727764507 | United Kingdom | European Union |
| 168 | Cyprus Mail | http://www.cyprus-mail.com/ | 308790590111 | Cyprus | European Union |
| 169 | Cyprus Traveller | http://cyprustraveller.com/ | 363334110438460 | Cyprus | European Union |
| 170 | The Daily Caller | http://dailycaller.com/ | 182919686769 | USA | North America |
| 171 | The Daily and Sunday Express | http://www.express.co.uk/ | 129617873765147 | United Kingdom | European Union |
| 172 | Daily Finance | http://www.dailyfinance.com/ | 65750045740 | USA | North America |
| 173 | Independent Newspapers Nigeria | http://dailyindependentnig.com/ | 344063115645237 | Nigeria | Africa |
| 174 | Daily Mail | https://www.dailymail.co.uk/home/index.html | 164305410295882 | United Kingdom | European Union |
| 175 | Daily Maverick | http://www.dailymaverick.co.za/ | 171090380838 | South Africa | Africa |
| 176 | The Mirror Online | http://www.mirror.co.uk/ | 6149699161 | United Kingdom | European Union |
| 177 | Daily Mirror | http://www.dailymirror.lk/ | 129843276524 | Sri Lanka | Asia |
| 178 | Daily Monitor | http://www.monitor.co.ug/ | 105583497196 | Uganda | Africa |
| 179 | Daily Nation | http://www.nation.co.ke/ | 105983259496 | Kenya | Africa |
| 180 | The Daily Nation | http://zambiadailynation.com | 520665064730150 | Zambia | Africa |
| 181 | Daily News | http://www.dailynews.co.zw/ | 129826597051376 | Zimbabwe | Africa |
| 182 | The Daily Observer | http://observer.gm/ | 155754624520664 | Gambia | Africa |
| 183 | Daily Post Nigeria | http://dailypost.ng/ | 247156892008627 | Nigeria | Africa |
| 184 | The Daily Star - Lebanon | http://www.dailystar.com.lb/News/Lebanon-News.ashx#axzz2hm2EvBhu | 43930085355 | Lebanon | Middle East |
| 185 | The Daily Star | http://www.thedailystar.net/ | 100117146754830 | Bangladesh | Asia |
| 186 | Daily Star Nigeria | http://dailystar.com.ng/ | 475906012461633 | Nigeria | Africa |
| 187 | Daily Times | http://www.dailytimes.com.pk/ | 710854355609434 | Pakistan | Middle East |
| 188 | Daily Times of Nigeria | http://dailytimes.com.ng/ | 482749575105792 | Nigeria | Africa |
| 189 | Daily Trust | http://www.dailytrust.com.ng/ | 96160950863 | Nigeria | Africa |
| 190 | Dalje | http://dalje.com/en/ | 161625400547781 | Croatia | European Union |
| 191 | The Dallas Morning News | http://www.dallasnews.com/ | 20946638799 | USA | North America |
| 192 | Dawn | http://dawn.com/ | 86398345441 | Pakistan | Middle East |
| 193 | Dayton Daily News | http://www.daytondailynews.com/ | 168815400507 | USA | North America |
| 194 | DCist | http://dcist.com/ | 258755510899692 | USA | North America |
| 195 | Deccan Herald | http://www.deccanherald.com/ | 318318083127 | India | Asia |
| 196 | Defense News | http://www.defensenews.com/ | 70531852030 | USA | North America |
| 197 | Dehai Eritrean News | http://www.dehai.org/ | 224118577679888 | Eritrea | Africa |
| 199 | The Denver Post | http://www.denverpost.com/ | 6181619439 | USA | North America |
| 200 | Derby Telegraph | http://www.thisisderbyshire.co.uk/ | 142370589115824 | United Kingdom | European Union |
| 201 | Derry Journal Newspaper | http://www.derryjournal.com/ | 104291312965731 | Ireland | European Union |
| 202 | The Des Moines Register | http://www.desmoinesregister.com/ | 8031989578 | USA | North America |
| 203 | Detroit Free Press | http://www.freep.com/ | 13642915529 | USA | North America |
| 204 | Deutsche Welle | http://www.dw.de/ | 24369314439 | Germany | European Union |
| 205 | Diamond Development Initiative | http://www.ddiglobal.org/media/ | 118224424879645 | United Kingdom | European Union |
| 206 | The Israeli Diamond Industry | http://www.israelidiamond.co.il/english/index.aspx | 215033265188341 | Israel | Middle East |
| 207 | Digital Journal | http://digitaljournal.com/ | 67175109350 | USA | North America |
| 208 | Digjitale | http://digjitale.com | 247722835253293 | France | European Union |
| 209 | DNA India | http://www.dnaindia.com/ | 154284380440 | India | Asia |
| 210 | Dominican Today | http://www.dominicantoday.com/ | 154894428290 | Domenican Republic | Central America |
| 211 | Donegal Democrat | http://www.donegaldemocrat.ie/ | 112739298752474 | Ireland | European Union |
| 212 | Defence and Security Alert | http://www.dsalert.org/ | 197617058395 | India | Asia |
| 213 | Dublin People | http://www.dublinpeople.com/ | 144107362283722 | Ireland | European Union |
| 214 | Dundalk Democrat | http://www.dundalkdemocrat.ie/ | 62703437305 | Ireland | European Union |
| 215 | Dunmow Broadcast | http://www.dunmowbroadcast.co.uk/home/ | 181182540669 | United Kingdom | European Union |
| 216 | DutchNews | http://www.dutchnews.nl/ | 314156250291 | Netherlands | European Union |
| 217 | East Anglian Daily Times | http://www.eadt.co.uk/home | 6478299951 | United Kingdom | European Union |
| 218 | In2EastAfrica | http://in2eastafrica.net/ | 179227395457307 | Tanzania | Africa |
| 219 | Earthquake Report | http://earthquake-report.com/ | 145927268753491 | Canada | North America |
| 220 | European Asylum Support Office | https://easo.europa.eu/ | 1449052665318446 | Malta | European Union |
| 221 | ECHO - Humanitarian Aid and Civil Protection | http://ec.europa.eu/echo/news/index\_en.htm | 146955527905 | Belgium | European Union |
| 222 | eCanada Now | http://www.ecanadanow.com/ | 270230853108003 | Canada | North America |
| 223 | Eco-Business | http://www.eco-business.com/ | 136176823413276 | Singapore | Asia |
| 224 | The Economic Times | http://www.economictimes.com/ | 21540067693 | India | Asia |
| 225 | Edmonton Journal | http://www.edmontonjournal.com/index.html | 100429659640 | Canada | North America |
| 226 | Egypt Independent | http://www.egyptindependent.com/ | 244154415645700 | Egypt | Global |
| 227 | 89.7 Bay Network | http://www.bay.com.mt/ | 186470373746 | Malta | European Union |
| 228 | EITI - Extractive Industries Transparency Initiative | https://eiti.org/ | 182289264273 | Norway | European Other |
| 229 | Kathimerini English Edition | http://www.ekathimerini.com/ | 142088502518488 | Greece | European Union |
| 230 | Elections Canada | http://www.elections.ca/home.aspx | 633812813408377 | Canada | North America |
| 231 | Electoral Commission of Jamaica | http://www.eoj.com.jm/ | 229947173716650 | Jamaica | North America |
| 232 | El Pa�s | http://elpais.com/elpais/inenglish.html | 279465748829565 | Spain | European Union |
| 233 | Executive Mansion - Government the Republic of Liberia | http://www.emansion.gov.lr/ | 310591779028211 | Liberia | Africa |
| 234 | Emirates 247 | http://www.emirates247.com/ | 136509933034734 | USA | North America |
| 235 | Trend News Agency | http://en.trend.az/ | 187192942493 | Azerbaijan | Asia |
| 236 | eNCA - e-News Channel Africa | http://www.enca.com | 160836574053016 | South Africa | Africa |
| 237 | EnergyWorld Magazine | http://www.energyworldmag.com/ | 201095326580070 | Romania | Global |
| 238 | Engadget | http://www.engadget.com/ | 5738237369 | USA | North America |
| 239 | The Chosun Ilbo | http://english.chosun.com/ | 129892740363306 | South Korea | Asia |
| 240 | KBS World Radio | http://world.kbs.co.kr/english/ | 170517169668683 | South Korea | Asia |
| 241 | Enough Project | http://www.enoughproject.org/blog | 252083412738 | USA | North America |
| 242 | Cincinnati Enquirer | http://www.cincinnati.com/ | 36808884697 | USA | North America |
| 243 | European Policy Centre | http://www.epc.eu/ | 115769411816768 | Belgium | European Union |
| 244 | EPP Group | http://www.eppgroup.eu/home/en/default.asp | 291699857688 | Belgium | European Union |
| 245 | El Paso Times | http://www.elpasotimes.com/ | 115612465136194 | Mexico | North America |
| 246 | ERR News | http://news.err.ee/ | 147712425255536 | Estonia | European Union |
| 247 | The Express Tribune | http://tribune.com.pk | 111457038871331 | Pakistan | Asia |
| 248 | EUbusiness | http://www.eubusiness.com/ | 215108901846669 | United Kingdom | European Union |
| 249 | Council of the European Union | http://www.consilium.europa.eu/en/homepage/ | 147547541961576 | Belgium | European Union |
| 250 | EUobserver | http://www.euobserver.com/ | 227715390929 | Belgium | European Union |
| 251 | EurActiv | http://www.euractiv.com/en/ | 15299247059 | United Kingdom | European Union |
| 252 | EurasiaNet | http://www.eurasianet.org | 61048477840 | USA | North America |
| 253 | Eurasia Review | http://www.eurasiareview.com/ | 339585248573 | USA | North America |
| 254 | EU Reporter | http://www.eureporter.co/ | 172730922764856 | Belgium | European Union |
| 255 | Euromoney | http://www.euromoney.com | 192279900885723 | United Kingdom | European Union |
| 256 | Euronews | http://www.euronews.com/ | 101402598109 | France | European Union |
| 257 | European Commission | http://europa.eu/rapid/search.htm | 107898832590939 | Belgium | European Union |
| 258 | European Railway Review | http://www.europeanrailwayreview.com/ | 404359882930504 | United Kingdom | European Union |
| 259 | ESA - European Space Agency | http://www.esa.int/ESA | 54912575666 | Netherlands | European Union |
| 260 | ESRF - European Synchrotron Radiation Facility | http://www.esrf.eu/home.html | 116961611670251 | France | European Union |
| 261 | Europe's World | http://europesworld.org/feed/ | 143299008908 | Belgium | European Union |
| 262 | EURweb | http://www.eurweb.com/ | 45752458150 | USA | North America |
| 263 | Evening Echo | http://www.eecho.ie/ | 137101942966920 | Ireland | European Union |
| 264 | London Evening Standard | http://www.standard.co.uk/ | 165348596842143 | United Kingdom | European Union |
| 265 | Expatica | http://www.expatica.com/uk | 206982432584 | United Kingdom | European Union |
| 266 | Trinidad Express | http://www.trinidadexpress.com/ | 134545155813 | Trinidad and Tobago | South America |
| 267 | Eyewitness News | http://ewn.co.za/ | 168892509821961 | South Africa | Africa |
| 268 | Pambazuka News | http://www.pambazuka.net/en/ | 210266369010114 | South Africa | Africa |
| 269 | FairWarning | http://www.fairwarning.org/ | 307820806867 | USA | North America |
| 270 | Famagusta Gazette | http://famagusta-gazette.com | 156070247783260 | Cyprus | European Union |
| 271 | Farmers Weekly | http://www.fwi.co.uk/ | 17050705902 | United Kingdom | European Union |
| 272 | Farming Life | http://www.farminglife.com/ | 243070359106664 | United Kingdom | European Union |
| 273 | Ferghana News | http://enews.fergananews.com/ | 140679709325246 | Russia | Asia |
| 274 | The Fiji Times Online | http://www.fijitimes.com/ | 7130088677 | Fiji | Oceania |
| 275 | Financial Mirror | http://www.financialmirror.com/ | 112107185515325 | Cyprus | European Union |
| 276 | Financial Times | http://www.ft.com/home/europe | 8860325749 | United Kingdom | European Union |
| 277 | Finland Times | http://www.finlandtimes.fi | 189776234502564 | Finland | Global |
| 278 | Fish Information and Services | http://fis.com/index.asp?l=e | 228637974386 | Japan | Asia |
| 279 | Fars News Agency in English | http://english.farsnews.com/ | 199676426877938 | Iran | Middle East |
| 280 | Focus Taiwan | http://focustaiwan.tw/ | 196543097045548 | Taiwan | Asia |
| 281 | Forbes | http://www.forbes.com/ | 30911162508 | USA | North America |
| 282 | Foreign Affairs | http://www.foreignaffairs.org/ | 35640742015 | USA | North America |
| 283 | 4-traders | http://www.4-traders.com/ | 570518562988663 | France | European Union |
| 284 | Fox Business | http://www.foxbusiness.com/index.html | 12795435237 | USA | North America |
| 285 | Fox News | http://www.foxnews.com/ | 15704546335 | USA | North America |
| 286 | France 24 | http://www.france24.com/en/ | 176585044433 | France | European Union |
| 287 | FriedlNews | http://www.friedlnews.com/ | 139471392786597 | Austria | European Union |
| 288 | FrontPage Magazine | http://frontpagemag.com/ | 296396490870 | USA | North America |
| 289 | This is Africa | http://www.thisisafricaonline.com/ | 779213412106756 | United Kingdom | European Union |
| 290 | Gabz-FM | http://www.gabzfm.com/ | 510261369119743 | Botswana | Africa |
| 291 | Gambia News Online | http://gambianewsonline.com | 108203649260707 | Gambia | Africa |
| 292 | The Gazette | http://thegazette.com/ | 7711594722 | Canada | North America |
| 293 | Geo News | http://www.geo.tv/Default.html | 101059326616167 | Pakistan | Asia |
| 294 | Georgia Today | http://www.georgiatoday.ge/ | 358827420882252 | Georgia | Asia |
| 295 | GhanaWeb | http://www.ghanaweb.com/ | 349823335095071 | Ghana | Africa |
| 296 | GINA - Government Information Agency | http://www.gina.gov.gy/home/ | 275385732567266 | Guyana | South America |
| 297 | Barbados Government Information Service | http://www.gisbarbados.gov.bb/ | 271334199698066 | Barbados | Central America |
| 298 | Gizmodo | http://gizmodo.com/ | 5718758966 | USA | North America |
| 299 | Jamaica Gleaner | http://jamaica-gleaner.com/ | 116087685213012 | Jamaica | North America |
| 300 | Global Issues | http://www.globalissues.org/ | 116446965860 | Haiti | Central America |
| 301 | GlobalPost | http://www.globalpost.com | 35930083446 | USA | North America |
| 302 | Global Research - Centre for Research on Globalization | http://www.globalresearch.ca/ | 200870816591393 | Canada | North America |
| 303 | Global Security | http://www.globalsecurity.org | 130707690322552 | USA | North America |
| 304 | Tax-News | http://www.tax-news.com/ | 375456009146619 | United Kingdom | European Union |
| 305 | Global Times | http://www.globaltimes.cn/index.html | 115591005188475 | China | Asia |
| 306 | Global Voices | http://globalvoicesonline.org/ | 6110663875 | Netherlands | European Union |
| 307 | The Boston Globe | http://www.bostonglobe.com/ | 5637143257 | USA | North America |
| 308 | INTA - International Trademark Association | http://www.inta.org | 132681243408881 | USA | North America |
| 309 | Government of Ghana | http://www.ghana.gov.gh/ | 395672803872128 | Ghana | Africa |
| 310 | South African Government | http://www.gov.za/ | 194109891221 | South Africa | Africa |
| 311 | Government of Prince Edward Island | http://www.gov.pe.ca/ | 122466107790811 | Canada | North America |
| 312 | Great Lakes Voice | http://greatlakesvoice.com/ | 123665291025561 | Ruanda | Africa |
| 313 | Greenpeace | http://www.greenpeace.org/international/en/ | 7297163299 | Netherlands | European Union |
| 314 | The Greens - European Free Alliance in the European Parliament | http://www.greens-efa.eu/ | 150527751674751 | Germany | European Union |
| 315 | Grenada Informer | http://www.thegrenadainformer.com/news | 444053475702112 | Grenada | Central America |
| 316 | Oxford International | http://www.oxfam.org/ | 197021747008439 | USA | North America |
| 317 | Pacific Daily News | http://www.guampdn.com/ | 90329609573 | Guam | Oceania |
| 318 | The Guardian | http://www.guardian.bz/ | 625050694259788 | Belize | North America |
| 319 | GUE/NGL - Confederal Group of the European United Left/Nordic Green Left | http://guengl.eu/group | 164355333623231 | Belgium | European Union |
| 320 | Gulf Daily News | http://www.gulf-daily-news.com/ | 382277502795 | Bahrain | Middle East |
| 321 | Gulf News | http://gulfnews.com/news | 143017562431177 | United Arab Emirates | Middle East |
| 322 | Gulf-Times | http://www.gulf-times.com/ | 274209762705885 | Qatar | Middle East |
| 323 | Gurtong Trust | http://www.gurtong.net/ | 105221526186803 | Sudan | Africa |
| 324 | Guyana Chronicle | http://www.guyanachronicle.com/ | 607416296042745 | Guyana | South America |
| 325 | Haaretz | http://www.haaretz.com/ | 64588666340 | Israel | Middle East |
| 326 | Haveeru | http://www.haveeru.com.mv/ | 1619525378288076 | Maldives | Asia |
| 327 | Hellenic Shipping News Worldwide | http://www.hellenicshippingnews.com/ | 336846839674977 | Greece | European Union |
| 328 | The Herald | http://www.herald.ie/ | 167882646558086 | Ireland | European Union |
| 329 | Herald Scotland | http://www.heraldscotland.com/news | 271154343382 | United Kingdom | European Union |
| 330 | The Daily Herald | https://www.thedailyherald.sx/ | 842786335748518 | Netherlands Antilles | North America |
| 331 | The Heritage Foundation | http://www.heritage.org/ | 21375324480 | USA | North America |
| 332 | Hiawatha World Online | http://www.hiawathaworldonline.com/ | 140225006013485 | USA | North America |
| 333 | Highland Radio | http://www.highlandradio.com/ | 104685286233423 | Ireland | European Union |
| 334 | Hindustan times | http://www.hindustantimes.com/ | 111445058579 | India | Asia |
| 335 | History News Network | http://historynewsnetwork.org/ | 187220577957886 | USA | North America |
| 336 | Homeland Security Newswire | http://homelandsecuritynewswire.com/ | 355645114549300 | USA | North America |
| 337 | HTS St. Lucia | http://www.htsstlucia.org/ | 110158905668844 | St. Lucia | Central America |
| 338 | The Huffington Post US | http://www.huffingtonpost.com/?country=US | 18468761129 | USA | North America |
| 340 | L'Humanit� | http://www.humaniteinenglish.com/ | 211118048927823 | France | European Union |
| 341 | Human Rights Watch | http://www.hrw.org/ | 42940254353 | USA | North America |
| 342 | H�rriyet Daily News | http://www.hurriyetdailynews.com/ | 353247165122 | Turkey | EU Candidate |
| 343 | IAEA - International Atomic Energy Agency | http://www.iaea.org | 96699782061 | Austria | European Union |
| 344 | iafrica | http://www.iafrica.com/ | 95285567334 | South Africa | Global |
| 345 | BIO - Biotechnology Innovation Organization | https://www.bio.org/ | 37791069588 | USA | North America |
| 346 | IBTimes - International Business Times US | http://www.ibtimes.com/ | 128026713884051 | USA | North America |
| 347 | IBTimes - International Business Times India | http://www.ibtimes.co.in/ | 134755750024728 | India | Asia |
| 348 | IBTimes - International Business Times Australia | http://au.ibtimes.com/ | 125955890795363 | Australia | Oceania |
| 349 | IBTimes - International Business Times UK | http://www.ibtimes.co.uk/ | 224377357631653 | United Kingdom | European Union |
| 350 | IceNews | http://www.icenews.is | 147438545270363 | Iceland | European Other |
| 351 | International Consortium of Investigative Journalists | http://www.icij.org/ | 99609391511 | USA | North America |
| 352 | International Catholic Migration Mission | http://www.icmc.net/ | 124780317689542 | Switzerland | European Other |
| 353 | International Committee of the Red Cross | http://www.icrc.org/eng | 336620687262 | Switzerland | European Other |
| 354 | IFEX | http://www.ifex.org | 164693013619204 | Tunisia | Africa |
| 355 | Intergovernmental Authority on Development | http://www.igad.org/ | 247268655431305 | Algeria | Africa |
| 356 | IHS Janes's 360 | http://www.janes.com/products/janes/index.aspx | 118688984881608 | United Kingdom | European Union |
| 357 | Africa Review | http://www.africareview.com/ | 265970097056 | Kenya | Africa |
| 358 | IMEC | http://www2.imec.be/be\_en/home.html | 48799938690 | Belgium | European Union |
| 359 | International Monetary Fund | http://www.imf.org/external/index.htm | 629825573770523 | USA | North America |
| 360 | IMPEL | http://impel.eu/ | 1554126881544220 | Belgium | European Union |
| 361 | In-Cyprus | http://www.incyprus.com.cy/ | 160324974064965 | Cyprus | European Union |
| 362 | Independent.ie | http://www.independent.ie | 96796398469 | Ireland | European Union |
| 363 | The Independent | http://www.independent.co.ug/ | 555291447866478 | Uganda | Africa |
| 364 | Reuters India | http://in.reuters.com | 27917365629 | India | Global |
| 365 | IndyStar | http://www.indystar.com/ | 9812439851 | USA | North America |
| 366 | The India Exoress | http://www.expressindia.com/ | 163648403825 | India | Asia |
| 367 | InfoMine | http://www.infomine.com/ | 298200071890 | Canada | North America |
| 368 | Informant� | http://www.informante.web.na/ | 183476948385940 | Namibia | Africa |
| 369 | Inkatha Freedom Party | http://www.ifp.org.za/ | 1679769598931438 | South Africa | Africa |
| 370 | Inmarsat | http://www.inmarsat.com/ | 317156988374684 | United Kingdom | European Union |
| 371 | InSight Crime | http://www.insightcrime.org/ | 147437478639179 | Colombia | South America |
| 372 | Institute for War and Peace Reporting | http://iwpr.net/ | 27283822672 | USA | North America |
| 373 | Institut Laue-Langevin | http://www.ill.eu/ | 148452651846298 | France | European Union |
| 374 | International Alert | http://www.international-alert.org/ | 23799263071 | United Kingdom | European Union |
| 375 | International Civic Aviation Organization | http://www.icao.int/Pages/default.aspx | 1509413712605916 | Canada | North America |
| 376 | International IDEA | http://www.idea.int/ | 196055247078923 | Sweden | European Union |
| 377 | Internews | http://www.internews.org | 54282384157 | USA | North America |
| 378 | Interpol | http://www.interpol.int/ | 282736625084940 | France | European Union |
| 379 | IntraFish | http://www.intrafish.no/fn/ | 117849188285395 | Norway | European Other |
| 380 | Investor's Business Daily | http://www.investors.com/default.htm | 18107421115 | USA | North America |
| 381 | IOL | http://www.iol.co.za/ | 21993963624 | South Africa | Africa |
| 382 | International Organization for Migration | http://www.iom.int/ | 161303029020 | Switzerland | European Other |
| 383 | International Peace Information Service | http://ipisresearch.be/publication-category/weekly-brief/ | 380249695352799 | Belgium | European Union |
| 384 | Inter Press Service | http://www.ipsnews.net/ | 41883361077 | Algeria | Africa |
| 385 | Iran Daily | http://www.iran-daily.com/ | 233108793428420 | Iran | Middle East |
| 386 | UN Integrated Regional Information Networks | http://www.irinnews.org/ | 49134323939 | USA | North America |
| 387 | Irish Abroad | http://www.irishabroad.com/Home/Default.aspx | 104716382899827 | Ireland | European Union |
| 388 | Irish Examiner | http://www.irishexaminer.com/ | 175322202485450 | Ireland | European Union |
| 389 | Irish Farmers Journal | http://www.farmersjournal.ie/ | 208480693834 | Ireland | European Union |
| 390 | Irish Sun | http://www.irishsun.com/ | 138617672855411 | Ireland | European Union |
| 391 | The Irish Times | http://www.irishtimes.com/ | 45441411157 | Ireland | European Union |
| 392 | Iran-Va-Jahan | http://iranvajahan.net/en/ | 1498587017035003 | Iran | Middle East |
| 393 | International Relations and Security Network | http://www.isn.ethz.ch/ | 141505796320 | Switzerland | European Other |
| 394 | Israel National News | http://www.israelnationalnews.com/ | 102510354100 | Israel | Middle East |
| 395 | Institute for Security Studies | http://www.issafrica.org/ | 204054676287947 | Algeria | Africa |
| 396 | ITV News | http://www.itv.com/news/ | 148007467671 | United Kingdom | European Union |
| 397 | Indian Television | http://www.indiantelevision.com/ | 517483194951446 | India | Asia |
| 398 | The Jakarta Post | http://www.thejakartapost.com/ | 102060486500863 | Indonesia | Asia |
| 399 | Jamaica Information Service | http://www.jis.gov.jm/ | 129366111456 | Jamaica | North America |
| 400 | The Jamaica Observer | http://www.jamaicaobserver.com/ | 95746458800 | Jamaica | North America |
| 401 | Jamaica Star | http://jamaica-star.com/ | 94678696229 | Jamaica | North America |
| 402 | Japan Today | http://www.japantoday.com/ | 206382352722433 | Japan | Asia |
| 403 | Japan Update | http://www.japanupdate.com/ | 193676320645525 | Japan | Asia |
| 404 | JCK - Jewelry Industry News - Trends and Analysis | http://www.jckonline.com/ | 72079325608 | USA | North America |
| 405 | Journal of Commerce | http://www.joc.com/ | 73172116398 | USA | North America |
| 406 | Jollof News | http://www.jollofnews.com/ | 264329360246554 | Gambia | Africa |
| 407 | Journal Sentinel | http://www.jsonline.com/ | 16511263815 | USA | North America |
| 408 | Justice in Mexico | http://justiceinmexico.org/ | 204554056223931 | Mexico | North America |
| 409 | Kaieteur News | http://www.kaieteurnewsonline.com/ | 126977184000816 | Guyana | South America |
| 410 | Kansas City Star | http://www.kansascity.com/ | 81580834093 | USA | North America |
| 411 | KCLR 96 FM | http://kclr96fm.com/ | 146296768437 | Ireland | European Union |
| 412 | Khaleej Times | http://www.khaleejtimes.com/ | 211983032863 | United Arab Emirates | Middle East |
| 413 | Kilkenny People | http://www.kilkennypeople.ie/ | 148324858612545 | Ireland | European Union |
| 414 | KIMT News | http://kimt.com/ | 89871599854 | USA | North America |
| 415 | Kippreport | http://www.kippreport.com/ | 115801885129398 | United Arab Emirates | Middle East |
| 416 | Korea.net | http://www.korea.net/news/news\_main.html | 181274814520 | South Korea | Asia |
| 417 | Korea JoongAng Daily | http://koreajoongangdaily.joinsmsn.com/ | 190108377775039 | South Korea | Asia |
| 418 | Krakow Post | http://www.krakowpost.com/ | 82857764783 | Poland | European Union |
| 420 | KUAM News | http://www.kuam.com/ | 39567613741 | Guam | Oceania |
| 421 | Kuensel | http://www.kuenselonline.com/ | 140918395943068 | Bhutan | Asia |
| 422 | Ekurd Daily | http://www.ekurd.net/ | 299681230060006 | United Kingdom | European Union |
| 423 | Kuwait Times | http://news.kuwaittimes.net/website/ | 80073822064 | Kuwait | Middle East |
| 424 | Kyiv Post | http://www.kyivpost.com/ | 218630044858401 | Ukraine | European Other |
| 425 | The Labour Spokesman | http://www.labourspokesman.com/ | 376337209232404 | Saint Kitts and Nevis | Central America |
| 426 | La Crosse Tribune | http://lacrossetribune.com/ | 66774556378 | USA | North America |
| 427 | Los Angeles Daily News | http://www.dailynews.com/ | 55107646487 | USA | North America |
| 428 | Latin American Herald Tribune | http://www.laht.com/index.asp | 198630266978389 | Venezuela | South America |
| 429 | Colombo Page | http://www.colombopage.com | 164230806937385 | Sri Lanka | Asia |
| 430 | La Strada International | http://lastradainternational.org/?main=home | 107095159376651 | Netherlands | European Union |
| 431 | Los Angeles Times | http://www.latimes.com | 5863113009 | Saudi Arabia | Asia |
| 432 | Las Cruces Sun-News | http://www.lcsun-news.com/ | 148251148521543 | USA | North America |
| 433 | Leinster Leader | http://www.leinsterleader.ie/ | 445456162136874 | Ireland | European Union |
| 434 | Leitrim Observer | http://www.leitrimobserver.ie/ | 223377501087566 | Ireland | European Union |
| 435 | Lesotho Times | http://lestimes.com/ | 263874940420105 | Lesotho | Africa |
| 436 | Limerick Leader | http://www.limerickleader.ie/ | 242208309132671 | Ireland | European Union |
| 437 | Limerick's Live 95FM | http://www.live95fm.ie/ | 102779203091115 | Ireland | European Union |
| 438 | Lincoln Journal Star | http://journalstar.com/ | 12493817252 | USA | North America |
| 439 | LMFM 95.8 | http://www.lmfm.ie/Home | 185885781448435 | Ireland | European Union |
| 440 | Longford Leader | http://www.longfordleader.ie/ | 342214075801273 | Ireland | European Union |
| 441 | Lydian International | http://www.lydianinternational.co.uk/ | 186900121339682 | United Kingdom | European Union |
| 442 | Ma'an News | http://www.maannews.net/en/ | 66674009455 | Israel | Middle East |
| 443 | Mail&Guardian Online | http://mg.co.za/ | 161428670566653 | South Africa | Africa |
| 444 | Malaysiakini | http://www.malaysiakini.com/ | 47298465905 | Malaysia | Asia |
| 445 | Maldives Independent | http://maldivesindependent.com/ | 295327362366 | Maldives | Asia |
| 446 | Malta Star | http://www.maltastar.com/ | 456049707755135 | Malta | European Union |
| 447 | Malta Today | http://www.maltatoday.com.mt | 21535456940 | Malta | European Union |
| 448 | Managing Intellectual Property | http://www.managingip.com/ | 133642376683214 | USA | North America |
| 449 | Manica Post | http://www.manicapost.com/ | 152535288239280 | Zimbabwe | Africa |
| 450 | The Maravi Post | http://www.maravipost.com/ | 145297758853971 | Malawi | Africa |
| 451 | Marine Link | http://www.marinelink.com/ | 283894011630279 | USA | North America |
| 452 | Market Watch | http://www.marketwatch.com/ | 131043201847 | USA | North America |
| 453 | McClatchy DC | http://www.mcclatchydc.com/ | 27177163800 | USA | North America |
| 454 | Meath Chronicle | http://www.meathchronicle.ie/ | 107690035817 | Ireland | European Union |
| 455 | Media Monitoring Africa | http://www.mediamonitoringafrica.org/ | 247186148894 | South Africa | Africa |
| 456 | MercoPress | http://en.mercopress.com/ | 139220750082 | Brazil | South America |
| 457 | The Mercury News | http://www.mercurynews.com/ | 63095136336 | USA | North America |
| 458 | The Messenger | http://www.messenger.com.ge/ | 155667667914073 | Georgia | Asia |
| 459 | Messenger-Inquirer | http://www.messenger-inquirer.com/ | 127426175386 | Cayman Islands | North America |
| 460 | Metro �ireann | http://metroeireann.com/ | 183768441657310 | Ireland | European Union |
| 461 | Metro | http://metro.co.uk/ | 117118184990145 | United Kingdom | European Union |
| 462 | Miami Herald | http://www.miamiherald.com/ | 38925837299 | USA | North America |
| 463 | Mid-Day | http://www.mid-day.com/ | 101982830708 | India | Asia |
| 464 | Midlands 103 | http://www.midlandsradio.fm/ | 101869576532842 | Ireland | European Union |
| 465 | The Midland Tribune | http://www.midlandtribune.ie/ | 106650586336938 | Ireland | European Union |
| 466 | Midwest Radio | http://www.midwestradio.ie/ | 115609018461867 | Ireland | European Union |
| 467 | The Milli Gazette | http://www.milligazette.com | 108910729126586 | India | Asia |
| 468 | Mindanao Examiner | http://www.mindanaoexaminer.com/ | 197032943653361 | Philippines | Asia |
| 469 | Mineweb | http://www.mineweb.com/ | 133899146653638 | South Africa | Africa |
| 470 | MINING.com | http://www.mining.com/ | 170783659631727 | Canada | North America |
| 471 | Mining News | http://www.miningnews.net/ | 145316085622369 | Australia | Oceania |
| 472 | Mining Technology | http://www.mining-technology.com/ | 326019370778750 | United Kingdom | European Union |
| 473 | Mining Weekly | http://www.miningweekly.com/ | 112406545484603 | South Africa | Africa |
| 474 | The Government of the Bahamas | http://www.bahamas.gov.bs/ | 361099777333587 | Bahamas | Central America |
| 475 | Mizzima | http://www.mizzima.com/ | 150773224985493 | India | Asia |
| 476 | Mmegi Online | http://www.mmegi.bw/ | 121039846823 | Botswana | Africa |
| 477 | MNI News | https://mninews.marketnews.com/ | 124576787245 | USA | North America |
| 478 | Modern Ghana | http://www.modernghana.com/ | 366939671198 | USA | North America |
| 479 | InfoPak - Ministry of Information | http://www.infopak.gov.pk/ | 712150158816432 | Pakistan | Middle East |
| 480 | Le Monde Diplomatique | http://mondediplo.com/ | 198560474895 | France | European Union |
| 481 | Mondo Visione | http://www.mondovisione.com/ | 169767016460715 | United Kingdom | European Union |
| 482 | MoneyWeek | http://moneyweek.com/ | 110326662354766 | United Kingdom | European Union |
| 483 | Monsters and Critics | http://www.monstersandcritics.com/ | 193326863118 | United Kingdom | European Union |
| 484 | Montreal Gazette | http://www.montrealgazette.com/ | 273805206181 | Canada | North America |
| 485 | Morningstar | http://www.morningstar.com/ | 428809707142935 | USA | North America |
| 486 | The Moscow Times | http://www.themoscowtimes.com/ | 203688324765 | Russia | Asia |
| 487 | Dayton Most Metro | http://mostmetro.com/ | 216346418455890 | USA | North America |
| 488 | MPR News | http://minnesota.publicradio.org/features/ | 99142348590 | USA | North America |
| 489 | Doctors Without Borders | http://www.msf.org/topics/mediterranean-migration | 33110852384 | Switzerland | European Other |
| 490 | MSNBC | http://www.msnbc.com/ | 273864989376427 | USA | North America |
| 491 | Muscatine Journal | http://muscatinejournal.com/ | 55294244167 | USA | North America |
| 492 | Marianas Variety | http://www.mvariety.com/ | 266082993493810 | Nepal | Oceania |
| 493 | Joy Online | http://news.myjoyonline.com/ | 115080018529590 | Ghana | Africa |
| 494 | Naharnet | http://www.naharnet.com/ | 7227980682 | Lebanon | Middle East |
| 495 | Namibia Economist | http://www.economist.com.na/ | 538225062922656 | Namibia | Africa |
| 496 | NASA | https://www.nasa.gov/ | 54971236771 | USA | North America |
| 497 | National Mirror | http://nationalmirroronline.net/ | 296803303684069 | Nigeria | Africa |
| 498 | The Nation Barbados | http://www.nationnews.com/ | 270969375988 | Barbados | Central America |
| 499 | Natural Gas Europe | http://www.naturalgaseurope.com/ | 1425903867642047 | Canada | North America |
| 500 | NBC News | http://www.nbcnews.com/ | 155869377766434 | USA | North America |
| 501 | NBC Radio St Vincent and the Grenadines | http://www.nbcsvg.com/ | 119263408099925 | Saint Vincent and the Grenadines | Central America |
| 502 | New Civil Engineer | http://www.nce.co.uk/ | 166793706822441 | United Kingdom | European Union |
| 503 | Nepal News | http://www.nepalnews.com/ | 195499967173234 | Nepal | Asia |
| 504 | New Economics Foundation | http://www.neweconomics.org/ | 110275553302 | United Kingdom | European Union |
| 505 | New Era | http://www.newera.com.na/ | 131485450303317 | Namibia | Africa |
| 506 | New Europe | http://neurope.eu/ | 111051292072 | Netherlands | European Union |
| 507 | New Poland Express | http://www.newpolandexpress.pl/ | 164066290543 | Poland | European Union |
| 508 | news.com.au | http://www.news.com.au/ | 111416688885713 | Australia | Oceania |
| 509 | Kenya Today | http://www.kenya-today.com/ | 293248774116017 | Kenya | Africa |
| 510 | news24.com | http://www.news24.com/ | 10227041841 | South Africa | Africa |
| 511 | News 5 Belize | http://edition.channel5belize.com/ | 472013519586191 | Belize | North America |
| 512 | NEWS.am | http://news.am/eng/ | 160111214030709 | Armenia | Asia |
| 513 | NewsBlogged | http://newsblogged.com/ | 253819121296131 | USA | North America |
| 514 | NewsChannel5 | http://www.newschannel5.com/ | 91345192547 | United Kingdom | European Union |
| 515 | New Scientist | http://www.newscientist.com/ | 235877164588 | United Kingdom | European Union |
| 516 | NewsDay | https://www.newsday.co.zw/ | 215170571826981 | Zimbabwe | Africa |
| 517 | Durham Region | http://www.durhamregion.com/durhamregion/ | 115912925116255 | USA | North America |
| 518 | News from Africa | http://www.newsfromafrica.org/ | 157591600945944 | Kenya | Africa |
| 519 | The News-Gazette | http://www.news-gazette.com/ | 120713712889 | Canada | North America |
| 520 | Newstalk | http://www.newstalk.ie/ | 70425092906 | Ireland | European Union |
| 521 | Newstalk ZB | http://www.newstalkzb.co.nz/ | 171762839533606 | New Zealand | Oceania |
| 522 | New Statesman | http://www.newstatesman.com/ | 100959719644 | United Kingdom | European Union |
| 523 | Thanh Nien News | http://www.thanhniennews.com/Pages/default.aspx | 108754382559490 | Vietnam | Asia |
| 524 | Daily Newswatch | http://www.mynewswatchtimesng.com/ | 469757943065522 | Nigeria | Africa |
| 525 | New Zimbabwe | http://www.newzimbabwe.com/ | 125973854117415 | Zimbabwe | Africa |
| 526 | The Guardian | http://ngrguardiannews.com/ | 176502461635 | Nigeria | Africa |
| 527 | Business News | http://businessnews.com.ng/ | 211032168937402 | Nigeria | Africa |
| 528 | Masterweb Reports | http://nigeriamasterweb.com/Masterweb/ | 291985020925887 | Nigeria | Africa |
| 529 | The Nigerian Observer | http://www.nigerianobservernews.com/ | 148046368615205 | Nigeria | Africa |
| 530 | Nippon News | http://www.nipponnews.net/ | 277898815655 | Japan | Asia |
| 531 | The Times-Picayune | http://www.nola.com/ | 99597577059 | USA | North America |
| 532 | The North Africa Journal | http://north-africa.com/ | 110960715505 | USA | North America |
| 533 | The Northern Miner | http://www.northernminer.com/ | 276490482462006 | Canada | North America |
| 534 | The Northern Standard | http://northernstandard.ie/ | 168433759858012 | Ireland | European Union |
| 535 | Noseweek | http://www.noseweek.co.za/ | 116905531677008 | South Africa | Africa |
| 536 | NOW. | https://now.mmedia.me/lb/en | 171247176237894 | Lebanon | Middle East |
| 537 | NPR | http://www.npr.org | 10643211755 | USA | North America |
| 538 | Natural Resource Governance Institute | http://www.resourcegovernance.org/ | 219848484745333 | USA | North America |
| 539 | Nova Scotia | http://novascotia.ca/ | 161817467263 | Canada | North America |
| 540 | New Straits Times | http://www.nst.com.my/ | 135289458465 | Malaysia | Asia |
| 541 | Nyasa Times | http://www.nyasatimes.com/ | 250647473519 | Malawi | Africa |
| 542 | NY Daily News | http://www.nydailynews.com/ | 268914272540 | USA | North America |
| 543 | New York Post | http://nypost.com/ | 134486075205 | USA | North America |
| 544 | The New York Times | http://www.nytimes.com/ | 5281959998 | USA | North America |
| 545 | NZ Herald | http://www.nzherald.co.nz | 34497296301 | New Zealand | Oceania |
| 546 | The Daily Observer | http://antiguaobserver.com/ | 126646997376452 | Antigua and Barbuda | Central America |
| 547 | Ocean FM | http://oceanfm.ie/ | 115071508505532 | Ireland | European Union |
| 548 | Nuclear Energy Agency | http://www.oecd-nea.org/ | 161503327202946 | France | European Union |
| 549 | Offaly Express | http://www.offalyexpress.ie/ | 207799065511 | Ireland | European Union |
| 550 | Offaly Independent | http://www.offalyindependent.ie/ | 121886488053 | Ireland | European Union |
| 551 | The Spectator | http://www.spectator.co.uk/ | 111263798903232 | United Kingdom | European Union |
| 552 | Olu Famous | http://www.olufamous.com/ | 292824824087658 | Nigeria | Africa |
| 553 | Jamaicans.com | http://www.jamaicans.com/ | 128078147433 | Jamaica | North America |
| 554 | OneWorld | http://oneworld.org/ | 106968052697581 | United Kingdom | European Union |
| 555 | Government of Ontario | http://news.ontario.ca/newsroom/en | 367410119963157 | Canada | North America |
| 556 | Online Nigeria | http://www.onlinenigeria.com/uk/ | 143914392334873 | Nigeria | Africa |
| 557 | OpEdNews.com | http://www.opednews.com/ | 200583909956972 | USA | North America |
| 558 | Open Europe Today | http://openeurope.org.uk/ | 321253057971308 | United Kingdom | European Union |
| 559 | Organisation of Islamic Cooperation | http://www.oic-oci.org/home.asp | 176887235707491 | Palestine | Middle East |
| 560 | Organization for Security and Co-operation in Europe | http://www.osce.org/ | 59678478979 | Austria | European Union |
| 561 | Ovation International | http://ovationinternational.com/ | 58902011402 | Ghana | Africa |
| 562 | Oxford Analytica | http://www.oxan.com | 160525917321265 | United Kingdom | European Union |
| 563 | Pacific Islands Report | http://pidp.eastwestcenter.org/pireport/ | 184980038221527 | USA | North America |
| 564 | Pakistan Tribune | http://www.pakistantribune.com.pk/ | 556680544487686 | Pakistan | Middle East |
| 565 | Pakistan Observer | http://pakobserver.net/ | 143465663100 | Pakistan | Middle East |
| 566 | The Palestinian Information Centre | http://english.palinfo.com/site/pages/ | 233417303344412 | Palestine | Middle East |
| 567 | Pan European Networks | http://www.paneuropeannetworks.com/ | 230201663697109 | United Kingdom | European Union |
| 568 | The Patriot Post | http://patriotpost.us/ | 51560645913 | USA | North America |
| 569 | People's Daily | http://english.peopledaily.com.cn/ | 188625661189259 | China | Asia |
| 570 | People's Daily Online | http://www.peoplesdaily-online.com/ | 228444807376 | Nigeria | Africa |
| 571 | People's Review | http://www.peoplesreview.com.np/ | 191277000903079 | Nepal | Asia |
| 572 | The Phnom Penh Post | http://www.phnompenhpost.com/ | 154245617928723 | Cambodia | Asia |
| 573 | Phys | http://phys.org/ | 47849178041 | USA | North America |
| 574 | Twin Cities | http://www.twincities.com/ | 276896490404 | USA | North America |
| 575 | Pittsburgh Post-Gazette | http://www.post-gazette.com/ | 184142654825 | USA | North America |
| 576 | Planet Tonga | http://www.planet-tonga.com/ | 335939203143941 | Tonga | Oceania |
| 577 | POLITICO | http://www.politico.com/ | 62317591679 | USA | North America |
| 578 | POLITICO Europe | http://www.politico.eu/ | 587266261407195 | Belgium | European Union |
| 579 | Portfolio | http://www.portfolio.hu/en/ | 546626485410734 | Hungary | European Union |
| 580 | The Portugal News | http://www.theportugalnews.com/ | 322352061097 | Portugal | European Union |
| 581 | Marianas Variety Guam | http://mvguam.com/ | 269007167849 | Guam | Oceania |
| 582 | The Post | http://www.postzambia.com | 1512926715599838 | Zambia | Africa |
| 583 | Prague Daily Monitor | http://www.praguemonitor.com/ | 299876763374822 | Czech Republic | European Union |
| 584 | The Prague Post | http://www.praguepost.com/ | 128602892226 | Czech Republic | European Union |
| 585 | The Presidency of The Republic of South Africa | http://www.thepresidency.gov.za/ | 8383028996 | South Africa | Africa |
| 586 | PR-Inside | http://www.pr-inside.com/ | 161831927209456 | Austria | European Union |
| 587 | PR Newswire | http://www.prnewswire.com/ | 26247320522 | USA | North America |
| 588 | PTV World | http://ptvworldnews.com.pk | 406940679374569 | Pakistan | Middle East |
| 589 | Publish What You Pay | http://www.publishwhatyoupay.org/ | 176624229034172 | United Kingdom | European Union |
| 590 | The Punch | http://www.punchng.com/ | 206270189411151 | Nigeria | Africa |
| 591 | Rabble | http://rabble.ca/ | 115517676270 | Canada | North America |
| 592 | American Renaissance | http://www.amren.com/ | 19051993499 | USA | North America |
| 593 | Radio Australia | http://www.radioaustralia.net.au/international/ | 128522530498035 | Australia | Oceania |
| 594 | Dabanga | https://www.dabangasudan.org/en | 82615736748 | Sudan | Africa |
| 595 | RNZ International | http://www.rnzi.com | 7759768730 | New Zealand | Oceania |
| 596 | Radio Poland | http://www.thenews.pl/ | 121677301182864 | Poland | European Union |
| 597 | Radio Prague | http://www.radio.cz/en | 183496134175 | Czech Republic | European Union |
| 598 | Radio Caribbean International | http://www.rcistlucia.com/home/default.aspx | 386558457078 | St. Lucia | Central America |
| 599 | Red Pepper | http://www.redpepper.co.ug/ | 131452373539377 | Uganda | Africa |
| 600 | ReliefWeb | http://reliefweb.int/ | 57397818992 | Switzerland | European Other |
| 601 | Repeating Islands | http://repeatingislands.com/ | 928983033797008 | USA | North America |
| 602 | Reporter 365 | http://reporter365.com/ | 278327162277225 | USA | North America |
| 603 | Sahara Reporters | http://saharareporters.com/ | 96184337702 | Nigeria | Africa |
| 604 | Investing News Network | http://investingnews.com/ | 66284580629 | Canada | North America |
| 605 | Reuters | http://www.reuters.com/ | 114050161948682 | United Kingdom | European Union |
| 606 | Reuters UK | http://uk.reuters.com/ | 208314602512037 | United Kingdom | European Union |
| 607 | The Reykjav�k Grapevine | http://grapevine.is/ | 8969907778 | Iceland | European Other |
| 608 | Radio Free Europe/Radio Liberty | http://www.rferl.org/ | 122264309574 | Czech Republic | European Union |
| 609 | Radio France International | http://www.english.rfi.fr/ | 28764872018 | France | European Union |
| 610 | RNW Media | https://www.rnw.org/ | 42251765664 | Netherlands | European Union |
| 611 | Routes Online | http://www.routesonline.com/ | 126251777434574 | United Kingdom | European Union |
| 612 | Radio St. Lucia 97 | http://rslonline.com/ | 204248159604926 | St. Lucia | Central America |
| 613 | RT� News | http://www.rte.ie/ | 257558294273180 | Ireland | European Union |
| 614 | Russia Today | http://rt.com/ | 326683984410 | Russia | Asia |
| 615 | RTT News | http://www.rttnews.com/ | 166456801229 | USA | North America |
| 616 | Rwandinfo | http://rwandinfo.com/eng/ | 107926999225610 | Ruanda | Africa |
| 617 | The Salt Lake Tribune | http://www.sltrib.com/ | 35281584398 | USA | North America |
| 618 | Samoa News | http://www.samoanews.com/ | 446910992109668 | Samoa | Oceania |
| 619 | Samoa Observer | http://www.samoaobserver.ws/ | 278391252189695 | Northern Marianas | Oceania |
| 620 | Security Assistance Monitor | http://www.securityassistance.org/ | 134409343259972 | USA | North America |
| 621 | Syrian Arab News Agency | http://www.sana.sy/en/ | 166379316760326 | Syria | Middle East |
| 622 | The San Diego Union-Tribune | http://www.utsandiego.com/ | 133508396112 | USA | North America |
| 623 | South Africa News | http://www.sanews.gov.za/ | 612864685431270 | South Africa | Africa |
| 624 | The San Pedro Sun | http://www.sanpedrosun.com/ | 246187688749182 | Belize | North America |
| 625 | Saudi Gazette | http://www.saudigazette.com.sa/ | 137192489702943 | USA | North America |
| 626 | Save the Children | http://www.savethechildren.org/site/c.8rKLIXMGIpI4E/b.6115947/k.8D6E/Official\_Site.htm | 8047221596 | USA | North America |
| 627 | Santa Barbara Independent | http://www.independent.com/ | 30883294835 | USA | North America |
| 628 | SBS News | http://www.sbs.com.au/news/ | 125982670754724 | Australia | Oceania |
| 629 | SciDev.Net | http://www.scidev.net/global/ | 109375082451665 | United Kingdom | European Union |
| 630 | Science Alert | http://www.sciencealert.com/ | 7557552517 | USA | North America |
| 631 | Science | http://www.sciencemag.org/ | 96191425588 | USA | North America |
| 632 | Scientific American | http://www.scientificamerican.com/ | 22297920245 | USA | North America |
| 633 | Searchlight | http://searchlight.vc/ | 152771864791320 | Saint Vincent and the Grenadines | Central America |
| 634 | Seatrade Maritime News | http://www.seatrade-global.com/ | 470795739645931 | United Kingdom | European Union |
| 635 | The Seattle Times | http://seattletimes.com/html/home/index.html | 38472826214 | USA | North America |
| 636 | Security Council Report | http://www.securitycouncilreport.org/ | 131442826906850 | USA | North America |
| 637 | The Sentinel | http://www.thisisstaffordshire.co.uk/ | 11878899813 | United Kingdom | European Union |
| 638 | SFGate | http://www.sfgate.com/ | 105702905593 | USA | North America |
| 639 | Shabait | http://www.shabait.com/index.php | 455303841165054 | Eritrea | Africa |
| 640 | Shannon Side | http://www.shannonside.ie/ | 109597305738451 | Ireland | European Union |
| 641 | ShippingWatch - English | http://shippingwatch.com/ | 606618229391196 | Denmark | European Union |
| 642 | Silicon Republic | http://www.siliconrepublic.com/ | 166431264240 | Ireland | European Union |
| 643 | Sioux City Journal | http://siouxcityjournal.com/ | 51119691219 | USA | North America |
| 644 | SIPRI | http://www.sipri.org/ | 309816241933 | Sweden | European Union |
| 645 | Sky News | http://news.sky.com/ | 164665060214766 | United Kingdom | European Union |
| 646 | Sligo Weekender | http://www.sligoweekender.ie | 139420522800336 | Ireland | European Union |
| 647 | The Smithville Herald | http://www.smithvilleherald.com/ | 79136059123 | USA | North America |
| 648 | Socialists and Democrats in the European Parliament | http://www.socialistsanddemocrats.eu/ | 127925800618165 | Belgium | European Union |
| 649 | Mining Engineering Online | http://me.smenet.org/ | 43749334577 | USA | North America |
| 650 | The Sofia Echo | http://www.sofiaecho.com/ | 538279649571477 | Bulgaria | European Union |
| 651 | Novinite | http://www.novinite.com/ | 59362904867 | Bulgaria | European Union |
| 652 | South China Morning Post | http://www.scmp.com/portal/site/SCMP/ | 355665009819 | China | Asia |
| 653 | SouthernMinn | http://www.southernminn.com/ | 376746812416329 | USA | North America |
| 654 | South Sudan News Agency | http://www.southsudannewsagency.com/ | 124568540913185 | Sudan | Africa |
| 655 | Asia One | http://www.asiaone.com/A1Home/A1Home.html | 121790674546188 | Singapore | Asia |
| 656 | SpiceIslander | http://spiceislander.com/ | 147014315348275 | Grenada | Central America |
| 657 | Spiegel | http://www.spiegel.de/international/ | 39205942284 | Germany | European Union |
| 658 | Sport Fishing | http://www.sportfishingmag.com/ | 14417384293 | USA | North America |
| 659 | Sputnik | http://en.rian.ru/ | 357990416180 | Russia | Asia |
| 660 | Stabroek News | http://www.stabroeknews.com/ | 130548565052 | Guyana | South America |
| 661 | Standard Digital | http://www.standardmedia.co.ke | 88201339429 | Kenya | Africa |
| 662 | Honolulu Star-Advertiser | http://www.staradvertiser.com/ | 112299605447935 | USA | North America |
| 663 | Star Tribune | http://www.startribune.com | 42739463017 | USA | North America |
| 664 | State House Uganda | http://www.statehouse.go.ug/ | 158559710878311 | Uganda | Africa |
| 665 | Stuff | http://www.stuff.co.nz/ | 21253884267 | New Zealand | Oceania |
| 666 | Sudanese Media Center | http://smc.sd/eng/ | 328263710712432 | Sudan | Africa |
| 667 | Sudan Tribune | http://www.sudantribune.com/ | 147157358639460 | Sudan | Africa |
| 668 | Sudan Vision Daily | http://news.sudanvisiondaily.com/ | 100247370113145 | Sudan | Africa |
| 669 | Sunday BusinessPost | http://www.businesspost.ie/ | 811773748881458 | Ireland | European Union |
| 670 | Sunday Standard | http://www.sundaystandard.info/ | 404354156357611 | Botswana | Africa |
| 671 | Sunday World | http://www.sundayworld.com/ | 175466346599 | Ireland | European Union |
| 672 | Survival | http://www.survivalinternational.org/ | 19668531552 | United Kingdom | European Union |
| 673 | Swazi Observer | http://www.observer.org.sz/ | 253248861365174 | Swaziland | Africa |
| 674 | swissinfo | http://www.swissinfo.org/ | 81049933496 | Switzerland | European Other |
| 675 | SW Radio Africa | http://swradioafrica.com/ | 105987640597 | Zimbabwe | Africa |
| 676 | The Sidney Morning Herald | http://www.smh.com.au | 104598631263 | Australia | Oceania |
| 677 | Taipei Times | http://www.taipeitimes.com | 210998785327 | Taiwan | Asia |
| 678 | Talk City 91.1 | https://talkcity91fm.wordpress.com/ | 133446376708654 | Trinidad and Tobago | South America |
| 679 | Radio Tamazuj | https://radiotamazuj.org/ | 298524290184928 | Sudan | Africa |
| 680 | TamilNet | http://www.tamilnet.com/ | 129476557086822 | Sri Lanka | Asia |
| 681 | Tampa Bay Times | http://www.tampabay.com/ | 9924394837 | USA | North America |
| 682 | The Afro News | http://www.theafronews.com | 115007838514142 | Canada | North America |
| 683 | TASS | http://en.itar-tass.com/ | 221338351211505 | Russia | Asia |
| 684 | TCPalm | http://www.tcpalm.com/ | 62811590881 | USA | North America |
| 685 | Teagasc | http://www.teagasc.ie/ | 124025717676271 | Ireland | European Union |
| 686 | TechCrunch | http://www.techcrunch.com/ | 8062627951 | USA | North America |
| 687 | MIT Technology Review | http://www.technologyreview.com/ | 17043549797 | USA | North America |
| 688 | Tehran Times | http://tehrantimes.com/ | 265237380156809 | Iran | Middle East |
| 689 | The Telegraph | http://www.telegraph.co.uk/ | 143666524748 | United Kingdom | European Union |
| 690 | Television Jamaica | http://www.televisionjamaica.com/ | 135996373112459 | Jamaica | North America |
| 691 | Intelligence and Terrorism Information Center | http://www.terrorism-info.org.il/site/home/default.asp | 228183363874247 | Israel | Middle East |
| 692 | The Africa Report | http://www.theafricareport.com/ | 10750083165 | France | European Union |
| 693 | The Anguillian | http://theanguillian.com/ | 108369529266234 | Anguilla | North America |
| 694 | Arab News | http://www.arabnews.com/ | 10250877124 | Canada | North America |
| 695 | The Athens News | http://www.athensnews.com/ | 94790271234 | USA | North America |
| 696 | The Atlantic | http://www.theatlantic.com/ | 29259828486 | Canada | North America |
| 697 | The Australian | http://www.theaustralian.com.au/ | 45388134977 | Australia | Oceania |
| 698 | The Bahama Journal | http://jonesbahamas.com/ | 234368746664613 | Bahamas | Central America |
| 699 | The Baltic Times | http://www.baltictimes.com/ | 224870291024597 | Latvia | European Union |
| 700 | The Brunei Times | http://www.bt.com.bn/ | 119608528105659 | Brunei | Asia |
| 701 | The Buffalo News | http://www.buffalonews.com/ | 181362508150 | USA | North America |
| 702 | The Business Times | http://www.businesstimes.com.sg/ | 288031731262011 | Singapore | Asia |
| 703 | The Charlotte Observer | http://www.charlotteobserver.com/ | 42580340317 | USA | North America |
| 704 | Chicago Sun-Times | http://chicago.suntimes.com/ | 47864940833 | USA | North America |
| 705 | The Citizen | http://www.thecitizen.co.tz/ | 234582629937266 | Tanzania | Africa |
| 706 | The Clare Champion | http://www.clarechampion.ie/ | 137725366369881 | Ireland | European Union |
| 707 | The Costa Rica News | http://thecostaricanews.com/ | 214498872385 | CostaRica | Central America |
| 708 | The Courier | http://www.thecourier.co.uk | 325681791214 | United Kingdom | European Union |
| 709 | The Daily Tribune | http://www.tribune.net.ph/ | 253124567195 | Philippines | Asia |
| 710 | The Dominican | http://www.thedominican.net/ | 102945123139643 | Domenican Republic | Central America |
| 711 | The EastAfrican | http://www.theeastafrican.co.ke/ | 155073814515056 | Kenya | Africa |
| 712 | The Economist | http://www.economist.com | 6013004059 | United Kingdom | European Union |
| 713 | The Financial Express | http://www.financialexpress.com/ | 157671354275436 | India | Asia |
| 714 | The Financial Gazette | http://www.financialgazette.co.zw/ | 288500807952079 | Zimbabwe | Africa |
| 715 | The Fishing Website | http://www.fishing.net.nz/ | 147044425346125 | New Zealand | Oceania |
| 716 | The Freeport News | http://freeport.nassauguardian.net/ | 46726844467 | Bahamas | Central America |
| 717 | Fremont Tribune | http://fremonttribune.com/ | 108066434972 | USA | North America |
| 718 | The Frontier Post | http://www.thefrontierpost.com | 125681824173980 | Pakistan | Middle East |
| 719 | The Globe and Mail | http://www.theglobeandmail.com | 140961138903 | Canada | North America |
| 720 | The Grio | http://thegrio.com/ | 75928194876 | USA | North America |
| 721 | The Guardian | http://www.theguardian.com/uk | 10513336322 | United Kingdom | European Union |
| 722 | Harborough Mail | http://www.harboroughmail.co.uk/ | 219817851378553 | United Kingdom | European Union |
| 723 | The Herald | http://www.herald.co.zw/ | 380838785328009 | Zimbabwe | Africa |
| 724 | The Himalayan Times | http://www.thehimalayantimes.com/ | 166920243347320 | Nepal | Asia |
| 725 | The Hindu | http://www.thehindu.com/ | 163974433696568 | India | Asia |
| 726 | The International Institute for Strategic Studies | https://www.iiss.org/ | 29840385993 | United Kingdom | European Union |
| 727 | The Independent | http://www.independent.co.uk/ | 13312631635 | United Kingdom | European Union |
| 728 | The Insider | http://www.insiderzim.com/ | 122033327855067 | Zimbabwe | Africa |
| 729 | The Irish World | http://www.theirishworld.com | 49341828952 | Ireland | European Union |
| 730 | The Irrawaddy | http://www.irrawaddy.org | 112882212089978 | Thailand | Asia |
| 731 | Jarkata Globe | http://thejakartaglobe.beritasatu.com/ | 26578392579 | Indonesia | Asia |
| 732 | The Jamestown Foundation | http://www.jamestown.org/ | 106920576037705 | Iraq | Middle East |
| 733 | The Japan Times | http://www.japantimes.co.jp/ | 6321018343 | Japan | Asia |
| 734 | The Jerusalem Post | http://www.jpost.com/ | 159050394216641 | Israel | Middle East |
| 735 | The Jordan Times | http://jordantimes.com/ | 68601553125 | Jordan | Middle East |
| 736 | TheJournal.ie | http://www.thejournal.ie | 137576076262825 | Ireland | European Union |
| 737 | Daily Post | http://www.kenyan-post.blogspot.it/ | 271776752878863 | Kenya | Africa |
| 738 | Kenya Star | http://www.kenyastar.com/ | 143728442343553 | Kenya | Africa |
| 739 | The Korea Times | http://www.koreatimes.co.kr/www/index.asp | 227456724028836 | South Korea | Asia |
| 740 | Herald Argus | http://www.heraldargus.com/ | 565599370262091 | USA | North America |
| 741 | The Leader | http://www.theleader.info/ | 238743816156394 | Spain | European Union |
| 742 | The Local Austria | http://www.thelocal.at/ | 1426552570931475 | Austria | European Union |
| 743 | The Local Denmark | http://www.thelocal.dk/ | 1433835800214625 | Denmark | European Union |
| 744 | The Local France | http://www.thelocal.fr/ | 258002227555924 | France | European Union |
| 745 | The Local Germany | http://www.thelocal.de/ | 214435206012 | Germany | European Union |
| 746 | The Local Italy | http://www.thelocal.it/ | 384349881653734 | Italy | European Union |
| 747 | The Local Norway | http://www.thelocal.no/ | 514495608666410 | Sweden | European Union |
| 748 | The Local Spain | http://www.thelocal.es/ | 181292335348466 | Spain | European Union |
| 749 | The Local Sweden | http://www.thelocal.se/ | 220353389618 | Sweden | European Union |
| 750 | The Local Switzerland | http://www.thelocal.ch/ | 381700821924600 | Sweden | European Union |
| 751 | The Malta Independent | http://www.independent.com.mt/ | 480288348662981 | Malta | European Union |
| 752 | The Manila Times | http://www.manilatimes.net/ | 111026632011 | Philippines | Asia |
| 753 | The Mayo News | http://www.mayonews.ie/ | 64555193331 | Ireland | European Union |
| 754 | The Middle East Media Research Institute | http://www.memrijttm.org/ | 14310874716 | USA | North America |
| 755 | The Montserrat Reporter | http://www.themontserratreporter.com/ | 203080105851 | Monserrat | North America |
| 756 | Mumbai Mirror | http://www.mumbaimirror.com/ | 114157215335068 | India | Asia |
| 757 | The Namibian | http://www.namibian.com.na/ | 284922901537221 | Namibia | Africa |
| 758 | The National | http://www.thenational.ae/ | 148788988477345 | United Arab Emirates | Middle East |
| 759 | The Nationalist | http://www.nationalist.ie/ | 189189374491255 | Ireland | European Union |
| 760 | The Nation | http://nation.com.pk/ | 57383301711 | Pakistan | Asia |
| 761 | The Nation | http://www.thenation.com/ | 7629206115 | USA | North America |
| 762 | The New Age | http://www.thenewage.co.za/ | 138238556209769 | South Africa | Africa |
| 763 | New Republic | http://www.newrepublic.com/ | 161419311535 | USA | North America |
| 764 | The News | http://www.thenews.com.pk | 131257086910180 | Pakistan | Middle East |
| 765 | The New Sudan Vision | http://www.newsudanvision.com/ | 105477586161630 | Sudan | Africa |
| 766 | The New Times | http://www.newtimes.co.rw/ | 301148803327544 | Ruanda | Africa |
| 767 | New Vision | http://www.newvision.co.ug | 329423169077 | Uganda | Africa |
| 768 | The Norway Post | http://www.norwaypost.no | 185753021499903 | Norway | European Other |
| 769 | Nottingham Post | http://www.thisisnottingham.co.uk/home | 309833935716287 | United Kingdom | European Union |
| 770 | The Nassau Guardian | http://www.thenassauguardian.com/ | 131498596889079 | Bahamas | Central America |
| 771 | The Oregonian | http://www.oregonlive.com/ | 6321831972 | USA | North America |
| 772 | Ottawa Citizen | http://ottawacitizen.com/ | 6533373917 | Canada | North America |
| 773 | The Palestine Chronicle | http://palestinechronicle.com/ | 302592203117857 | Palestine | Middle East |
| 774 | The Parliament | https://www.theparliamentmagazine.eu/ | 471876160233 | Belgium | European Union |
| 775 | The Peninsula | http://www.thepeninsulaqatar.com/ | 111063551581 | Qatar | Middle East |
| 776 | The Post | http://thepost.co.ls/ | 1151120021571319 | Lesotho | Africa |
| 777 | The Railway Magazine | http://www.railwaymagazine.co.uk/ | 135345903226042 | United Kingdom | European Union |
| 778 | The Reporter | http://www.reporter.bz/ | 236081746489917 | Belize | North America |
| 779 | The Republic | http://www.therepublic.com/ | 54499120758 | USA | North America |
| 780 | The Russian Navy | http://rusnavy.com | 110378652324684 | Russia | Asia |
| 781 | The Scotsman | http://www.scotsman.com/the-scotsman | 293226174987 | United Kingdom | European Union |
| 782 | Daily Record and Sunday Mail | http://www.dailyrecord.co.uk/ | 187523381277554 | United Kingdom | European Union |
| 783 | The Scottish Government | http://home.scotland.gov.uk/home | 200786289976224 | United Kingdom | European Union |
| 784 | The Slovak Spectator | http://spectator.sme.sk/ | 59260989565 | Slovakia | European Union |
| 785 | The Sofia Globe | http://sofiaglobe.com/ | 445561718804553 | Bulgaria | European Union |
| 786 | The Source | http://www.thesourceng.com/index.html | 416043501758998 | Nigeria | Africa |
| 787 | The Southern Star | http://www.southernstar.ie/Home/ | 310855455632145 | Ireland | European Union |
| 788 | The Standard | http://www.thestandard.com.hk | 140973382599494 | Hong Kong | Asia |
| 789 | The Standard | http://www.thestandard.co.zw/ | 103305193039107 | Zimbabwe | Africa |
| 790 | The Standard | http://thestandard.com.ph/ | 835114793210549 | Philippines | Asia |
| 791 | The Star | http://www.the-star.co.ke | 224865787558662 | Kenya | Africa |
| 792 | The Star | http://thestar.com.my/ | 11450527254 | Malaysia | Asia |
| 793 | The St. Kitts and Nevis Observer | http://www.thestkittsnevisobserver.com/ | 476828099098371 | Saint Kitts and Nevis | Central America |
| 794 | The Straits Times | http://straitstimes.asiaone.com/ | 129011692114 | Singapore | Asia |
| 795 | The Sun | http://www.thesun.co.uk/sol/homepage/ | 161385360554578 | United Kingdom | European Union |
| 796 | The Sun | http://sunnewsonline.com/new/ | 139709372821659 | Nigeria | Africa |
| 797 | The Vincentian | http://thevincentian.com/ | 370649629649050 | Saint Vincent and the Grenadines | Central America |
| 798 | The Vindicator | http://www.vindy.com/ | 77780874764 | USA | North America |
| 799 | The Visitor | http://www.thevisitor.co.uk/ | 68554461041 | United Kingdom | European Union |
| 800 | The Voice | http://www.thevoicebw.com/ | 202343559085 | Botswana | Africa |
| 801 | The Voice | http://www.thevoiceslu.com/ | 160460070659226 | St. Lucia | Central America |
| 802 | The Washington Times | http://www.washingtontimes.com/ | 35994014410 | USA | North America |
| 803 | The Weather Channel | http://climate.weather.com/ | 118071565920 | USA | North America |
| 804 | The Yemen Times | http://www.yementimes.com/ | 130936023624588 | Yemen | Middle East |
| 805 | The Zambezian | http://www.thezambezian.com/ | 117965765017340 | Botswana | Africa |
| 806 | The Zimbabweans | http://www.thezimbabwean.co.uk | 217203691648702 | Zimbabwe | Africa |
| 807 | The Zimbabwe Independent | http://www.theindependent.co.zw/ | 331288056957552 | Zimbabwe | Africa |
| 808 | The Zimbabwe Mail | http://www.thezimbabwemail.com/ | 467260923305980 | Zimbabwe | Africa |
| 809 | This Day | http://www.thisdaylive.com/ | 142936439094106 | Nigeria | Africa |
| 810 | Thomson Reuters Foundation | http://www.trust.org | 31301735406 | United Kingdom | European Union |
| 811 | Tico Times | http://www.ticotimes.net/ | 124823954224180 | CostaRica | Central America |
| 813 | The Sunday Times | http://www.thesundaytimes.co.uk/sto/ | 147384458624178 | United Kingdom | European Union |
| 814 | Times Colonist | http://www.timescolonist.com/ | 50465429712 | Canada | North America |
| 815 | Times Daily | http://www.timesdaily.com/ | 121598674610038 | USA | North America |
| 816 | Times LIVE | http://www.timeslive.co.za/ | 136956534616 | South Africa | Africa |
| 817 | The Times of India | http://timesofindia.indiatimes.com/ | 26781952138 | India | Asia |
| 818 | Times of Malta | http://www.timesofmalta.com/ | 160227208174 | Malta | European Union |
| 819 | Times Of Oman | http://www.timesofoman.com/ | 136413806438283 | Oman | Middle East |
| 820 | Times Of Swaziland | http://www.times.co.sz/ | 142322529190260 | Swaziland | Africa |
| 821 | Times of Zambia | http://www.times.co.zm | 312705315487393 | Zambia | Africa |
| 822 | Tipp FM | http://www.tippfm.com/ | 221599401189017 | Ireland | European Union |
| 823 | Tipperary Star | http://www.tipperarystar.ie/ | 138048782943147 | Ireland | European Union |
| 824 | Tobago News | http://www.thetobagonews.com/ | 335414633195243 | Trinidad and Tobago | South America |
| 825 | Today FM | http://www.todayfm.com/home.aspx | 36108807567 | Ireland | European Union |
| 826 | TODAY | http://www.todayonline.com/ | 147858757571 | Singapore | Asia |
| 827 | Today's Zaman | http://www.todayszaman.com/mainAction.action | 173873526003430 | Turkey | EU Candidate |
| 828 | Topix | http://www.topix.net/ | 19062931201 | Canada | North America |
| 829 | The Toronto Star | http://www.thestar.com/ | 184906186150 | Canada | North America |
| 830 | Toronto Sun | http://www.torontosun.com/ | 189526659635 | Canada | North America |
| 831 | TorrentFreak | http://torrentfreak.com/ | 9087497371 | Netherlands | European Union |
| 832 | Trade Arabia | http://www.tradearabia.com/ | 439634269411506 | Bahrain | Middle East |
| 833 | Trade Winds | http://www.tradewindsnews.com/ | 132240771277 | USA | North America |
| 834 | Trading Markets | http://www.tradingmarkets.com/ | 151413724902563 | USA | North America |
| 835 | TribLIVE | http://triblive.com/ | 55863814979 | USA | North America |
| 836 | Tribune242 | http://www.tribune242.com/ | 33564008678 | Bahamas | Central America |
| 837 | Nigerian Tribune | http://www.tribune.com.ng/ | 77912203638 | Nigeria | Africa |
| 838 | Trinidad and Tobago Newsday | http://newsday.co.tt/ | 311107925748485 | Trinidad and Tobago | South America |
| 839 | T&T Guardian | http://www.guardian.co.tt/ | 78081948066 | Trinidad and Tobago | South America |
| 840 | TV3 | http://www.tv3.ie/news.php | 100183534537 | Ireland | European Union |
| 841 | United Arab Emirates Interact | http://www.uaeinteract.com/ | 193093937374424 | United Kingdom | European Union |
| 842 | United Democratic Movement | http://udm.org.za/ | 494801983919865 | South Africa | Africa |
| 843 | The Observer | http://www.observer.ug/ | 267688186591432 | Uganda | Africa |
| 844 | UK Government | https://www.gov.uk/government/organisations | 408582579294175 | United Kingdom | European Union |
| 845 | MSN News | http://www.msn.com/en-gb/news | 358837740527 | United Kingdom | European Union |
| 846 | United Nations Mission in Darfur | http://unamid.unmissions.org/Default.aspx?tabid=888 | 164650630228650 | Sudan | Africa |
| 847 | UN Dispatch | http://www.undispatch.com/ | 6069582499 | USA | North America |
| 848 | United Nations in Azerbaijan | http://www.un-az.org/ | 281319152013943 | Azerbaijan | Asia |
| 849 | Food and Agriculture Organization of the United Nations | http://www.fao.org/home/en/ | 46370758585 | Italy | European Union |
| 850 | UNHCR The UN Refugee Agency | http://www.unhcr.org/cgi-bin/texis/vtx/home/ | 13204463437 | Switzerland | European Other |
| 851 | UNIAN | http://www.unian.info/ | 1476822619259011 | Ukraine | European Other |
| 852 | United Nations | http://www.un.org/en/ | 54779960819 | USA | North America |
| 853 | United Nations Mission in South Sudan | http://unmiss.unmissions.org/ | 160839527325060 | Sudan | Africa |
| 854 | United Press International | http://www.upi.com/ | 101911273177707 | USA | North America |
| 855 | UN Office for the Coordination of Humanitarian Affairs | http://www.unocha.org | 135156639833927 | USA | Global |
| 856 | UN Office on Drugs and Crime | http://www.unodc.org/unodc/index.html | 43559937330 | Austria | European Union |
| 857 | United Nations Radio | http://www.unmultimedia.org/radio/english/ | 235134190239 | USA | North America |
| 858 | The Finnish Institute of International Affairs | http://www.fiia.fi/en/#tab1 | 117351301649740 | Finland | European Union |
| 859 | The Post | http://www.postnewsline.com/ | 146662198720301 | Cameroon | Africa |
| 860 | US Agency for International Development | http://www.usaidlandtenure.net/ | 62690599685 | USA | North America |
| 861 | USA Today | http://www.usatoday.com/ | 13652355666 | USA | North America |
| 862 | US Department of State | http://www.state.gov/ | 15877306073 | USA | North America |
| 863 | US News and World Report | http://www.usnews.com/ | 5834919267 | USA | North America |
| 864 | UTV | http://www.u.tv/ | 115860925163321 | Ireland | European Union |
| 865 | Estonian Ministry of Foreign Affairs | http://vm.ee/en | 57904691979 | Estonia | European Union |
| 866 | The Vancouver Sun | http://www.vancouversun.com/index.html | 7116517082 | Canada | North America |
| 867 | Vanguard | http://www.vanguardngr.com/ | 135140476511057 | Nigeria | Africa |
| 868 | Vietnam News | http://vietnamnews.vn/ | 254068371381722 | Vietnam | Asia |
| 869 | Vibe Ghana | http://vibeghana.com/ | 102329089882316 | Ghana | Africa |
| 870 | viEUws | http://www.vieuws.eu/ | 117994898215654 | France | European Union |
| 871 | The Virgin Islands Daily News | http://virginislandsdailynews.com/ | 132196636944018 | Virgin Islands | North America |
| 872 | Voice of Barbados | http://www.vob929.com/ | 1511527652404540 | Barbados | Central America |
| 873 | Voice of America | http://www.voanews.com/english/news/ | 36235438073 | USA | North America |
| 874 | Voice of Russia | http://sputniknews.com/voiceofrussia/ | 842603639086940 | Russia | European Other |
| 875 | Flanders News | http://deredactie.be/cm/vrtnieuws.english | 212796021897 | Belgium | European Union |
| 876 | The Register | http://www.theregister.co.uk/ | 206419956048907 | United Kingdom | European Union |
| 877 | Wales Online | http://www.walesonline.co.uk/ | 21226447182 | United Kingdom | European Union |
| 878 | Emirate News Agency | http://www.wam.ae/en/home.html | 124222007613925 | United Arab Emirates | Middle East |
| 879 | Wandsworth Guardian | http://www.wandsworthguardian.co.uk/ | 113349742029506 | United Kingdom | European Union |
| 880 | Warsaw Business Journal | http://www.wbj.pl/ | 91558833313 | Poland | European Union |
| 881 | Washington Examiner | http://washingtonexaminer.com/ | 40656699159 | USA | North America |
| 882 | The Washington Post | http://www.washingtonpost.com | 6250307292 | USA | North America |
| 883 | Watching America | http://watchingamerica.com/WA/ | 118258794866306 | USA | North America |
| 884 | Waterford News and Star | http://www.waterford-news.ie/ | 120792148007585 | Ireland | European Union |
| 885 | WBUR | http://www.wbur.org/ | 9427513649 | USA | North America |
| 886 | Waterloo Cedar Falls Courier | http://wcfcourier.com/ | 212364145478265 | USA | North America |
| 887 | Blitz | http://www.weeklyblitz.net/ | 242506829624 | Bangladesh | Asia |
| 888 | Western People | http://www.westernpeople.ie/ | 192666647731 | Ireland | European Union |
| 889 | Western Telegraph | http://www.westerntelegraph.co.uk/ | 180521675319022 | United Kingdom | European Union |
| 890 | Independent Westmeath | http://www.westmeathindependent.ie | 251005360424 | Ireland | European Union |
| 891 | Wexford Echo | http://www.wexfordecho.ie/ | 111308662280849 | Ireland | European Union |
| 892 | The White House | http://www.whitehouse.gov/ | 63811549237 | USA | North America |
| 893 | Wired | http://www.wired.com/ | 19440638720 | USA | North America |
| 894 | World News | http://wn.com/ | 229101503845879 | United Kingdom | European Union |
| 895 | The World Bank | http://documents.worldbank.org/curated/en/home | 153371894688575 | USA | North America |
| 896 | Worldcrunch | http://www.worldcrunch.com/ | 271986174770 | Ireland | European Union |
| 897 | World Finance | http://www.worldfinance.com | 725598290845413 | USA | North America |
| 898 | World Fishing and Aquaculture | http://www.worldfishing.net/ | 552321618120006 | United Kingdom | European Union |
| 899 | UN World Food Programme | http://www.wfp.org | 28312410177 | Italy | European Union |
| 900 | Omaha World-Herald | http://www.omaha.com/ | 6445219629 | USA | North America |
| 901 | World Maritime News | http://www.worldmaritimenews.com/ | 309171835876244 | Netherlands | European Union |
| 902 | Luxemburger Wort | http://www.wort.lu/en | 174116812644146 | Luxemburg | European Union |
| 903 | The Wall Street Journal | http://online.wsj.com/europe | 8304333127 | USA | North America |
| 904 | WWF | http://www.panda.org/ | 20373776304 | Switzerland | European Other |
| 905 | WXOW | http://www.wxow.com/ | 231408475203 | USA | North America |
| 906 | China Xinhua News | http://www.xinhuanet.com/english/ | 338109312883186 | China | Asia |
| 907 | Yahoo! Indian News | https://in.news.yahoo.com/ | 131747896861126 | India | Asia |
| 908 | Yahoo! News | http://news.yahoo.com/ | 338028696036 | USA | North America |
| 909 | Ya Libnan | http://yalibnan.com/site/ | 793949857311259 | Lebanon | Middle East |
| 910 | Yemen Post | http://www.yemenpost.net/ | 500685219968202 | Yemen | Middle East |
| 911 | YLE | http://www.yle.fi/ | 192534820828660 | Finland | European Union |
| 912 | Ynetnews | http://www.ynetnews.com/home/0 | 129653250402500 | Israel | Middle East |
| 913 | The Yorkshire Post | http://www.yorkshirepost.co.uk/ | 316795048375439 | United Kingdom | European Union |
| 914 | Zambia Daily Mail | http://www.daily-mail.co.zm/ | 172219889538699 | Zambia | Africa |
| 915 | Zambian Watchdog | http://www.zambianwatchdog.com | 129987587052000 | Zambia | Africa |
| 916 | Zambia Reports | http://www.zambiareports.com | 208755685909131 | Zambia | Africa |
| 917 | Thomson Reuters Zawya | http://www.zawya.com/countries/ps/default.cfm?cc | 112684488765578 | Palestine | Middle East |
| 918 | Zodiak Online | http://zodiakmalawi.com/ | 120942427951729 | Malawi | Africa |
| 919 | ZDNet | http://www.zdnet.com/ | 5953112932 | USA | North America |
| 920 | Zero Hedge | http://www.zerohedge.com/ | 116467201763793 | Bulgaria | European Union |
| 921 | ZF English | http://www.zfenglish.com/ | 130883803643584 | Romania | European Union |
| 922 | Zimbabwe Broadcasting Corporation | http://www.zbc.co.zw | 126155317511807 | Zimbabwe | Africa |
| 923 | ZimEye | http://www.zimeye.org | 150154425045764 | Zimbabwe | Africa |
| 924 | ZNS Network | http://www.znsbahamas.com/ | 248934135146821 | Bahamas | Central America |
